# Glial response to hypoxia in mutants of NPAS1/3 homolog Trachealess through Wg signaling to modulate synaptic bouton organization

**DOI:** 10.1371/journal.pgen.1007980

**Published:** 2019-08-05

**Authors:** Pei-Yi Chen, Yi-Wei Tsai, Ying-Ju Cheng, Angela Giangrande, Cheng-Ting Chien

**Affiliations:** 1 Institute of Neuroscience, National Yang-Ming University, Taipei, Taiwan; 2 Institute of Molecular Biology, Academia Sinica, Taipei, Taiwan; 3 Institut de Génétique et de Biologie Moléculaire et Cellulaire, Illkirch, France; 4 Centre National de la Recherche Scientifique, UMR7104, Illkirch, France; 5 Institut National de la Santé et de la Recherche Médicale, Illkirch, France; 6 Université de Strasbourg, Illkirch, France; Duke-NUS Medical School, SINGAPORE

## Abstract

Synaptic structure and activity are sensitive to environmental alterations. Modulation of synaptic morphology and function is often induced by signals from glia. However, the process by which glia mediate synaptic responses to environmental perturbations such as hypoxia remains unknown. Here, we report that, in the mutant for Trachealess (Trh), the *Drosophila* homolog for NPAS1 and NPAS3, smaller synaptic boutons form clusters named satellite boutons appear at larval neuromuscular junctions (NMJs), which is induced by the reduction of internal oxygen levels due to defective tracheal branches. Thus, the satellite bouton phenotype in the *trh* mutant is suppressed by hyperoxia, and recapitulated in wild-type larvae raised under hypoxia. We further show that hypoxia-inducible factor (HIF)-1α/Similar (Sima) is critical in mediating hypoxia-induced satellite bouton formation. Sima upregulates the level of the Wnt/Wingless (Wg) signal in glia, leading to reorganized microtubule structures within presynaptic sites. Finally, hypoxia-induced satellite boutons maintain normal synaptic transmission at the NMJs, which is crucial for coordinated larval locomotion.

## Author summary

Oxygen is essential for animals to maintain their life such as growth, metabolism, responsiveness, and movement. It is therefore important to understand how animal cells trigger hypoxia response and adapt to hypoxia thereafter. Both mammalian vascular and insect tracheal branches are induced to enhance the oxygen delivery. However, the study of hypoxia response in the nervous system remains limited. In this study, we assess the morphology of *Drosophila* neuromuscular junctions (NMJs), a model system to study development and function of synapses, in two hypoxia conditions, one with raising wild-type larvae in hypoxia, and the other in the *trachealess* (*trh*) mutant in which the trachea is defective, causing insufficient oxygen supply. Interestingly, the glial processes, normally wrapping around the axons, invade into synaptic boutons of NMJs under hypoxia. Also, the hypoxia-induced factor Sima activates the Wg signal in glia and the secreted Wg signal reorganizes the synaptic boutons as a response to hypoxia. This synaptic bouton reorganization might maintain normal synaptic transmission and locomotion of larvae.

## Introduction

Animals need oxygen and food, not only to sustain life, but also for motility. In vertebrates, oxygen and nutrients are delivered through the vascular systems to organs and tissues throughout the body. To maintain proper nutrient and oxygen supply, and thus physiological functions, the vascular system is also highly coordinated with the nervous system during development. Indeed, the vascular and nervous systems resemble each other in terms of their anatomical structures and developmental processes [1]. In the brain, nerves and vessels, form close associations and are in physical contact through the third player astrocytes to form neurovascular units (NVU) [2]. Such organization is essential for controlling oxygen and glucose delivery through the blood vessels by neuronal activity, and this regulatory process is mediated through the coupled astrocytes [3]. However, some invertebrates lack the complex vascular systems [4]. In nematodes, oxygen is supplied simply by ambient diffusion to inner cells [5]. Insects such as *Drosophila* have evolved a prototype of the tracheal system to deliver oxygen and a primitive vascular system, the dorsal vessel, to facilitate nutrient delivery [6]. However, the physical association of nerves, trachea, and glial processes has also been demonstrated at the NMJs of adult *Drosophila* flight muscles [7].

Animals respond to changing oxygen levels by altering their oxygen delivery system. Insufficient oxygen levels (hypoxia) activate a broad range of genes to re-establish body homeostasis. One crucial regulator of these hypoxia-responsive genes is the sequence-specific DNA-binding transcription factor hypoxia inducible factor 1 (HIF-1) [8]. HIF-1 consists of α and β subunits that form heterodimers [9]. Whereas HIF-1β is expressed constitutively, HIF-1α protein levels are modulated by oxygen levels [10]. Under normal oxygen conditions (normoxia), oxygen-dependent prolyl hydroxylases (PHDs) catalyze hydroxylation of a conserved prolyl residue in the central oxygen-dependent degradation (ODD) domain of HIF-1α [11]. Hydroxylation of HIF-1α promotes interaction with Von Hippel Lindau (VHL), which is the substrate recognition subunit of the cullin2-based E3 ubiquitin ligase, leading to HIF-1α ubiquitination and proteasomal degradation. Under hypoxia, prolyl hydroxylation does not occur, HIF-1α proteins are stabilized and are translocated from the cytoplasm to the nucleus where they form heterodimers with HIF-1β to activate transcription of target genes. One major class of target genes encoding the Fibroblast Growth Factor (FGF) is involved in inducing angiogenesis in mammals. In *Drosophila*, the FGF member encoded by *Branchless* (*Bnl*) induces tracheal branching [12]. When oxygen levels are reduced, oxygen-starved cells express Bnl as a chemo-attractant to guide the growth tracheal terminal branches toward them [13].

In addition to adaptations of the respiratory system, the nervous system also responds to hypoxia. Oxygen levels modulate the survival, proliferation, and differentiation of radial glial cells (RGCs) in the human cerebral cortex. Interestingly, physiological hypoxia (3% O_2_) induces neurogenesis and differentiation of RGCs into glutamatergic neurons [14]. Hypoxia induces neurite outgrowth in PC12 cells through activation of A2A receptor [15]. Brief exposure to anoxia and hypoglycemia caused axonal remodeling in hippocampal neurons, including presynaptic protrusion of filopodia and formation of multi-innervated spines [16]. Under hypoxia or upon depletion of PHD2, upregulation of the actin cross-linker Filamin A (FLNA) induces generation of more immature spines [17]. Astrocytes have been shown to play a crucial role in ischemic tolerance via the activation of P2X7 receptors, which trigger upregulation of HIF-1α [18].

Neuronal PAS (NPAS) proteins containing a DNA-binding Per-Arnt-Sim domain function in vascular and nervous system development. In mice, NPAS1 is responsible for cortical interneuron generation [19], whereas NPAS3 is required for adult neurogenesis [20]. NPAS1 and NPAS3 also play key roles in lung development [21, 22]. The homolog of NPAS1/3 in *Drosophila*, Trachealess (Trh), has been well studied for its involvement in formation of the respiratory tracheal system. Trh is a master regulator of tracheal cell fates, activating gene expression to induce tracheal development [23, 24]. However, the role of Trh in the development of other tissues, particularly the nervous system, is unknown. In this study, we found altered synaptic bouton morphology at the NMJs of *trh*^*1*^*/trh*^*2*^ mutants. By performing *trh-RNAi* knockdown and *UAS-trh* transgene rescue experiments, we show that *trh* is required in tracheal cells for normal bouton formation. Defective tracheal branching in the *trh*^*1*^*/trh*^*2*^ mutant mimics the effect of hypoxic conditions during larval development, and supplying higher than normal oxygen levels restored normal bouton morphology. We further show that glial cells respond to hypoxia by elevating Wnt/Wg expression to mediate synaptic bouton reorganization through HIF1-α/Sima in *Drosophila*. Finally, we reveal that this morphological change may be linked to normal synaptic transmission and locomotion of larvae.

## Results

### *trh* modulates synaptic bouton formation non-cell autonomously

**Fig 1:**
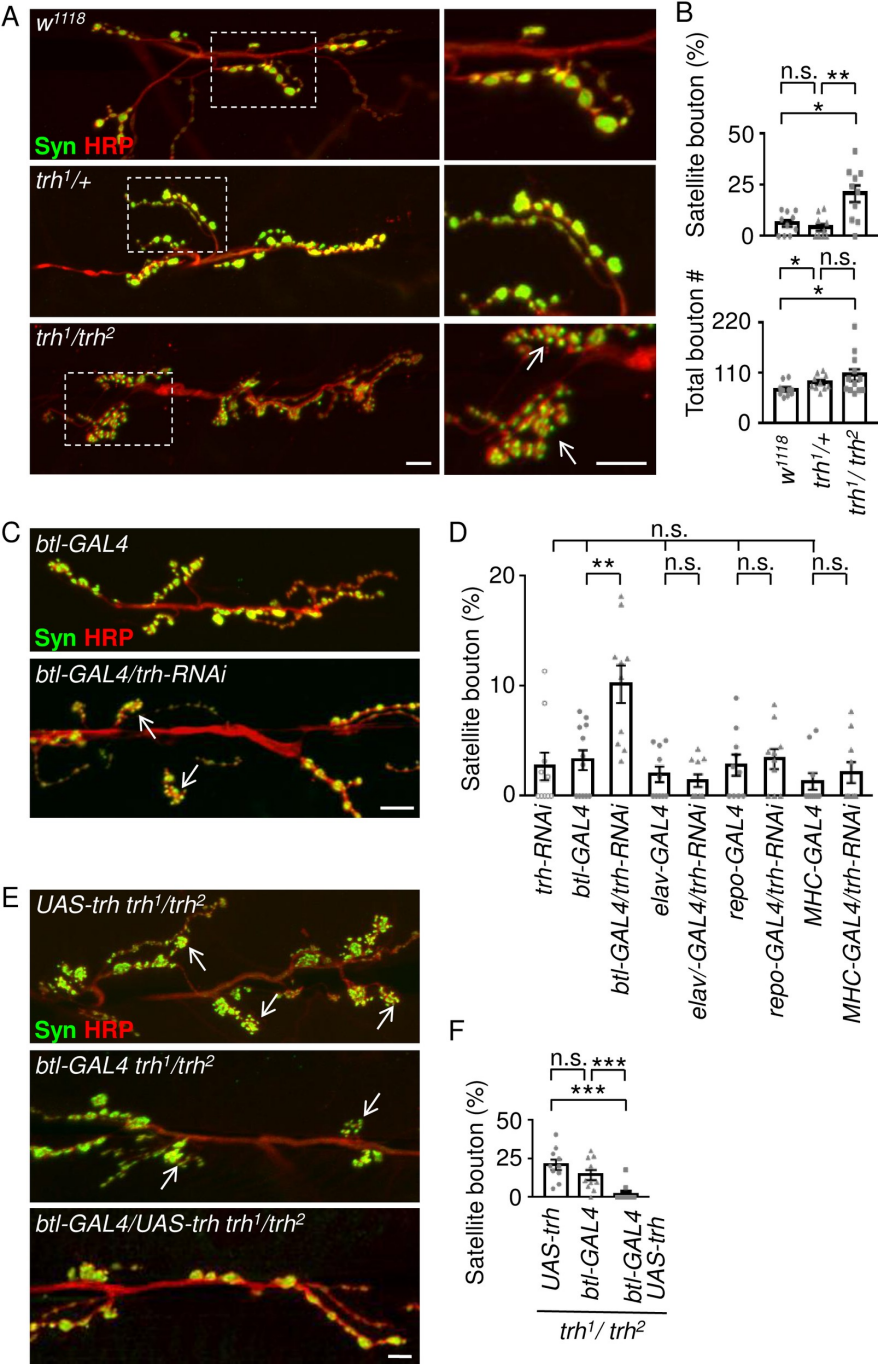
Satellite bouton formation at *trh* NMJs. (A, C, E) Images show NMJs of muscle 6/7 immunostaining for Syn (green) and HRP (red) in *w*^*1118*^, *trh*^*1*^*/+*, and *trh*^*1*^*/trh*^*2*^ (A), *btl-GAL4* and *btl*-*GAL4/trh-RNAi* (C), and *UAS-trh trh*^*1*^*/trh*^*2*^, *btl-GAL4 trh*^*1*^*/trh*^*2*^, and *btl-GAL4/UAS-trh trh*^*1*^*/trh*^*2*^ (E). Dashed squares in (A) are enlarged in right panels with white arrows indicating clusters of satellite boutons. Scale bars are 10 μm. (B, D, F) Bar graphs show mean ± standard error of mean for percentages of satellite boutons to total boutons (satellite + normal boutons)(B, upper graph, D, F) or total bouton number (B, bottom graph) for each genotype. Dots in each bar represent the distribution and number of data. Statistical significance by Mann-Whitney test is shown (n.s., no significance; *, p < 0.5; **, p < 0.01; ***, p < 0.001).

To understand the possible role of Trh in synapse formation, we examined NMJ morphology in the *trh* mutant. Since both *trh*^*1*^ and *trh*^*2*^ loss-of-function alleles are homozygous lethal [23, 25, 26], we examined the trans-heterozygous *trh*^*1*^*/trh*^*2*^ mutant that survived to adult stages and compared it to wild-type (*w*^*1118*^) and heterozygous *trh*^*1*^*/+* controls. Synaptic boutons of *w*^*1118*^ and *trh*^*1*^*/+* NMJs were evenly spaced along the axonal terminals, displaying the typical “beads-on-a-string” pattern (Fig 1A, upper and middle panels, enlarged images at right). Strikingly, the *trh*^*1*^*/trh*^*2*^ mutant larvae exhibited aberrant NMJ morphology. Small synaptic boutons formed clusters or surrounded a normal large bouton (Fig 1A, bottom panel); a phenotype described as “satellite boutons” [27]. This satellite bouton phenotype in the *trh*^*1*^*/trh*^*2*^ mutant was detected at a high frequency; 20.7 ± 4.1% (n = 10) of total boutons were satellite ones, more than three-fold increases compared to 4.3 ± 1.7% (n = 10) for *trh*^*1*^*/+* and 6.3 ± 1.7% (n = 10) for *w*^*1118*^ (Fig 1B). Although the percentage of satellite boutons in the *trh*^*1*^*/trh*^*2*^ mutant was greatly increased, the total bouton number was slightly higher than that observed in controls (74.1 ± 4.7, n = 10 in *w*^*1118*^; 89.6 ± 5.2, n = 10 in *trh*^*1*^*/+*; and 95.5 ± 9.1, n = 11 in *trh*^*1*^*/trh*^*2*^, bottom panel in Fig 1B). Also, the muscle areas were not significantly different from each other (S1A Fig). Given the small size and clustering of satellite boutons in the *trh*^*1*^*/trh*^*2*^ mutant, we examined whether these boutons express synaptic proteins normally. We found that the synaptic vesicle protein Synapsin (Syn in Fig 1A) was normally distributed relative to control, but the active zone protein Bruchpilot (Brp) was expressed at higher levels in the *trh*^*1*^*/trh*^*2*^ mutant (S1B Fig). The postsynaptic glutamate receptor, as revealed by GluRIII (S1B Fig) and GluRIIA (S1C Fig) signals, as well as dPAK (S1C Fig) were also localized in satellite boutons, which were surrounded by the subsynaptic reticulum protein Dlg (S1D Fig). Thus, although the Brp signal intensity in the *trh*^*1*^*/trh*^*2*^ mutant was stronger than in controls, the composition of synaptic proteins in satellite boutons was largely similar to that of normal-sized boutons.

As *trh* is expressed in both tracheal and nervous systems in embryonic stages [28], altered bouton morphology in the *trh*^*1*^*/trh*^*2*^ mutant could be due to a lack of *trh* in neurons, tracheal cells or other cells/tissues. Therefore, we performed *trh-RNAi* knockdown by using tissue-specific GAL4 drivers for trachea (*btl-GAL4*), neurons (*elav-GAL4*), glia (*repo-GAL4*), and muscles (*MHC-GAL4*). We observed a dramatic increase in satellite boutons upon tracheal *trh-RNAi* knockdown using *btl-GAL4* (10.2 ± 1.7%, n = 10) compared to those in the *trh-RNAi* (2.7 ± 1.3%, n = 10) or *btl-GAL4* (3.3 ± 0.9%, n = 12) control (Fig 1C and 1D. In contrast, *trh-RNAi* knockdown by other tissue-specific GAL4 drivers failed to significantly increase satellite boutons, displaying similar amounts of satellite boutons to respective GAL4 drivers (2.0 ± 0.7%, n = 10 in *elav-GAL4* v.s. 1.4 ± 0.6%, n = 10 in *elav-GAL4/trh-RNAi*; 2.8 ± 1.0%, n = 10 in *repo-GAL4* v.s. 3.4 ± 0.9%, n = 10 in *repo-GAL4*/*trh-RNAi*; and 1.3 ± 0.8%, n = 10 in *MHC-GAL4* v.s. 2.1 ± 1.0%, n = 10 in *MHC-GAL4/trh-RNAi*, Fig 1D).

To further confirm the necessity of tracheal *trh* for normal bouton formation, we performed a rescue experiment for the *trh*^*1*^*/trh*^*2*^ phenotype. When *UAS-trh* expression was driven by tracheal *btl-GAL4* in the *trh*^*1*^*/trh*^*2*^ mutant, the satellite bouton phenotype was suppressed (1.8 ± 1.4%, n = 13, Fig 1E and 1F). Controls bearing only *btl-GAL4* (14.3 ± 3.3%, n = 10) or *UAS-trh* (21.1 ± 3.3%, n = 10) still contained large amounts of satellite boutons. These results indicate that *trh* is required in the trachea for normal bouton formation.

### Hypoxia induces satellite bouton formation

Apart from specifying the tracheal cell fate, Trh is also involved in the branching of tubular structures during post-embryonic stages [24]. Therefore, we examined the tracheal phenotypes in the *trh*^*1*^*/trh*^*2*^ larvae (S2A Fig) and observed an increase in the number of terminal branches in the dorsal branch of the third segment (S2B Fig, 5.7 ± 0.15, n = 10 for *trh*^*1*^*/+*, and 7.5 ± 0.28, n = 11 for *trh*^*1*^*/trh*^*2*^). Furthermore, we identified morphological defects such as tracheal breaks and tangles, suggesting structural defects in the *trh*^*1*^*/trh*^*2*^ larvae (arrows in S2A Fig). Tracheal branching activity is enhanced under hypoxia [12]. Thus, the increased terminal branches in *trh*^*1*^*/trh*^*2*^ could be a compensatory mechanism for defective trachea formation.

**Fig 2:**
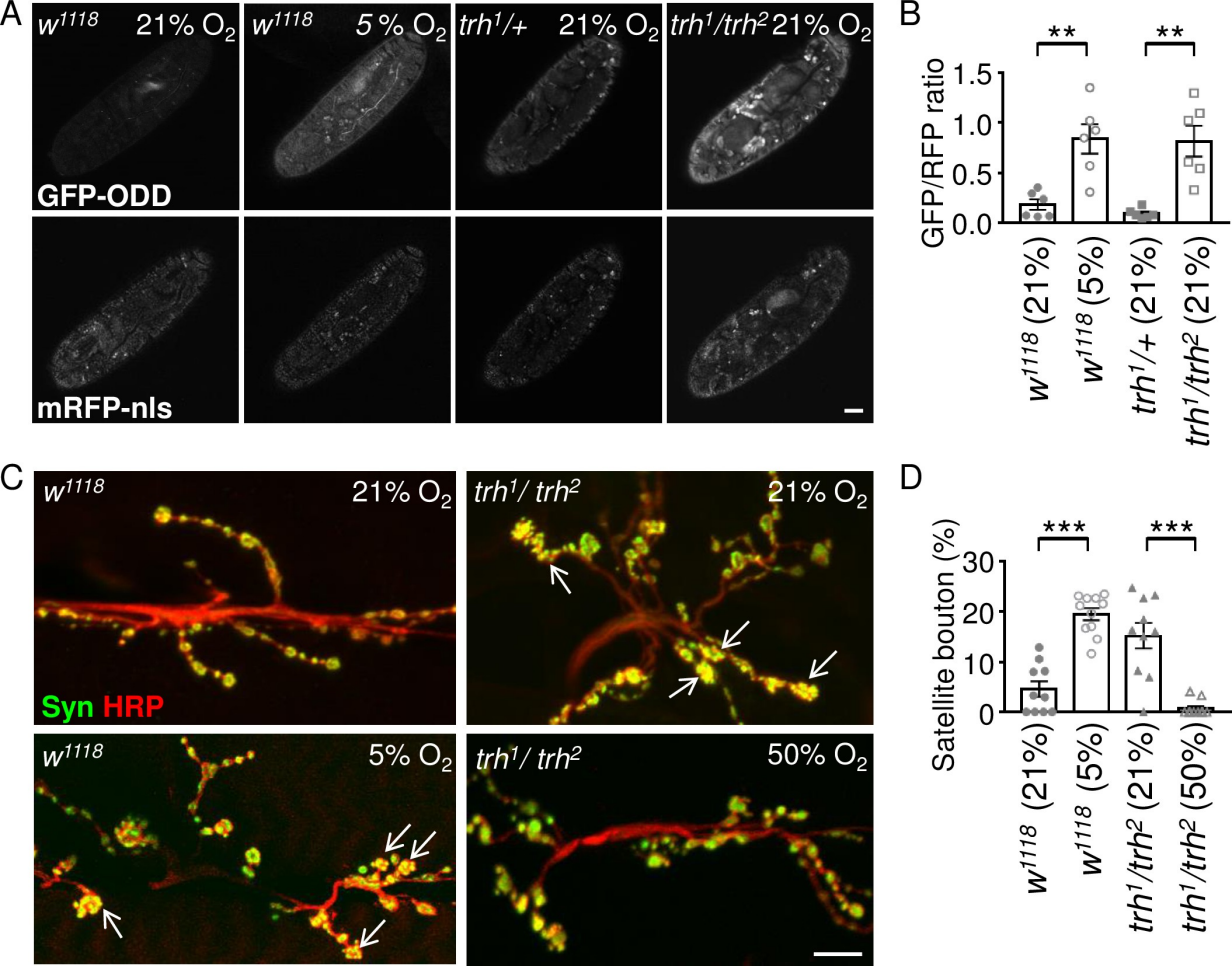
Induction of satellite boutons in *trh* mutant by hypoxia. (A) Images of late-stage embryos of *w*^*1118*^, *trh*^*1*^*/+*, and *trh*^*1*^*/trh*^*2*^ carrying the *GFP-ODD* reporter (top row) and *mRFP-nls* control (bottom row) under normoxia (21% O_2_) or hypoxia (5% O_2_). Scale bar represents 40 μm. (C) Images of NMJs from muscle 6/7 immunostained for Syn (green) and HRP (red) from *w*^*1118*^ in 21% or 5% O_2_ (left panels) and *trh*^*1*^*/trh*^*2*^ in 21% or 50% O_2_ (right panels). White arrows indicate clusters of satellite boutons. Scale bar represents 10 μm. (B, D) Bar graphs show averages of the GFP/RFP immunofluorescence intensity ratios (B), or percentages of satellite boutons to total boutons (D). Statistical significance assayed by Mann-Whitney test is shown (**, p < 0.01; ***, p < 0.001).

To understand whether *trh*^*1*^*/trh*^*2*^ mutant cells are under hypoxia, we used the hypoxia biosensor GFP-ODD, in which the GFP is fused to the ODD domain of Sima, under the control of the *ubiquitin-69E* (*ubi*) promoter [29]. We first confirmed that GFP-ODD signal was low under normoxia (21% O_2_) and enhanced under hypoxia (5% O_2_) in wild-type late-stage embryos when tracheal tubules are already formed and functioning [29]. Indeed, enhanced GFP signal was ubiquitous under hypoxia in wild-type embryos with some pronounced focal GFP signals (Fig 2A, upper row, and S2C Fig). The signal of mRFP-nls, also under the control of the *ubi* promoter as an internal control, remained constant under hypoxia (Fig 2A, bottom row, and S2D Fig). Quantification of the GFP/RFP ratio revealed a significant difference between normoxia and hypoxia (0.18 ± 0.05, n = 6 at 21% O_2_ and 0.84 ± 0.15, n = 6 at 5% O_2_, Fig 2B). We then examined whether oxygen supply is deficient in the *trh*^*1*^*/trh*^*2*^ mutant by measuring the GFP/RFP ratios. We detected a higher GFP/RFP ratio (0.82 ± 0.15, n = 6) in the mutant compared to that in heterozygous *trh*^*1*^*/+* (0.09 ± 0.02, n = 6) in the 21% O_2_ condition, supporting that the *trh*^*1*^*/trh*^*2*^ mutant senses reduced oxygen levels internally (Fig 2A and 2B).

Thus, formation of satellite boutons in the *trh*^*1*^*/trh*^*2*^ mutant could be caused by hypoxia. To test this hypothesis, we reared wild-type larvae under hypoxia (5%) and assessed synaptic bouton morphology at the third instar stage (Fig 2C). Consistently, small clustered boutons increased, mirroring the satellite bouton phenotype (Fig 2D, 4.6 ± 1.5%, n = 10 in 21% O_2_ v.s. 19.4 ± 1.2%, n = 11 in 5% O_2_). Furthermore, when the *trh*^*1*^*/trh*^*2*^ mutant was raised in a high oxygen level (50% O_2_), normal bouton morphology was restored (Fig 2C); the satellite bouton phenotype was almost completely suppressed (Fig 2D, 15.2 ± 2.5%, n = 10 in 21% O_2_ v.s. 0.74 ± 0.49%, n = 10 in 50% O_2_). These results suggest that hypoxia due to the defective tracheal system in the *trh*^*1*^*/trh*^*2*^ mutant induces the satellite bouton phenotype, and that this phenotype can be suppressed by extra oxygen supply.

### Glial HIF-1α/Sima mediates satellite bouton formation

**Fig 3:**
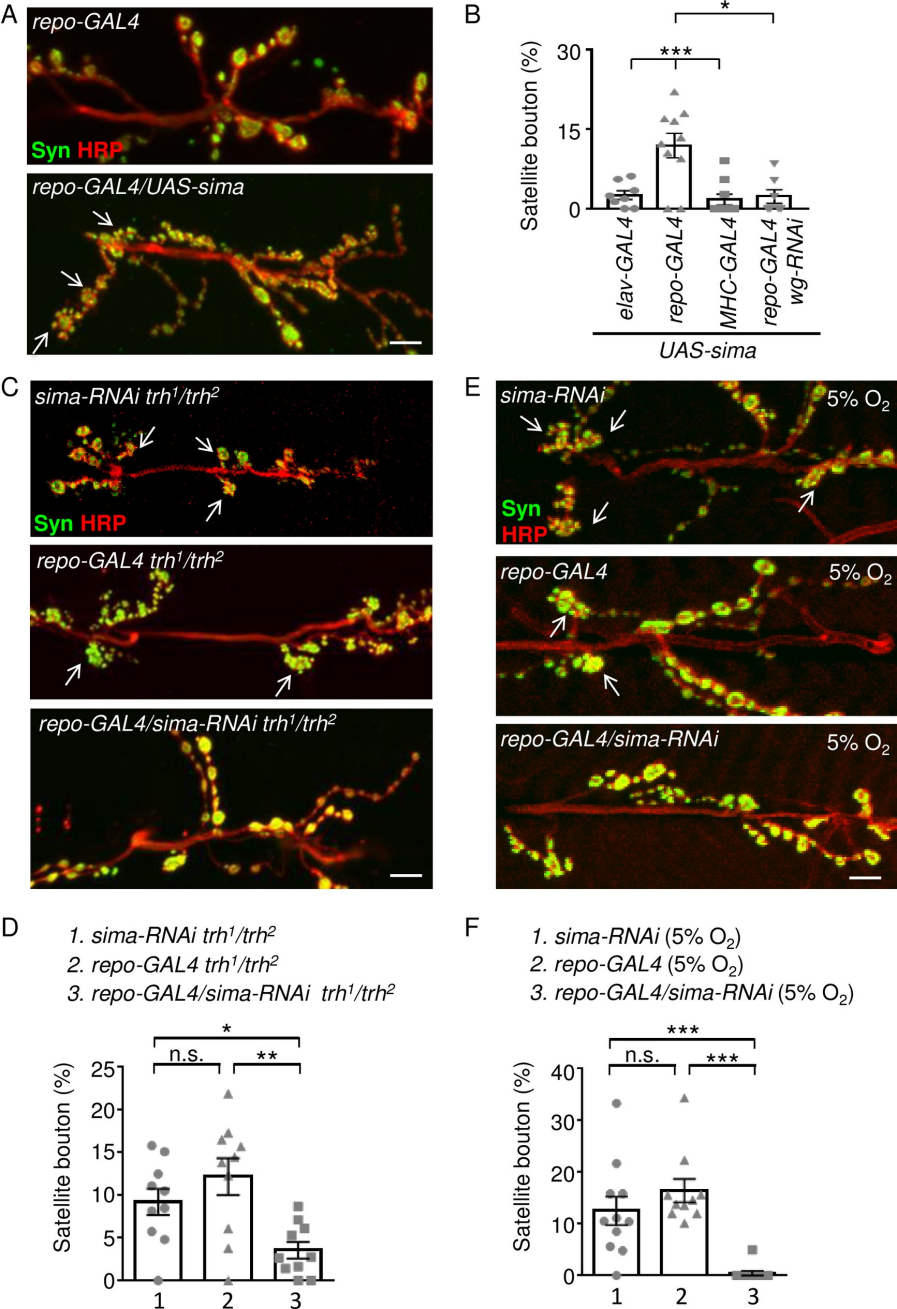
Satellite bouton induction by glial Sima in the *trh* mutant. (A, C, E) Images showing NMJs from muscle 6/7 immunostained for Syn (green) and HRP (red) in *repo-GAL4*, and *repo-GAL4/UAS-sima* (A), *sima-RNAi trh*^*1*^*/trh*^*2*^, *repo-GAL4 trh*^*1*^*/trh*^*2*^, and *repo-GAL4/sima-RNAi trh*^*1*^*/trh*^*2*^ (C), *sima-RNAi*, *repo-GAL4*, and *repo*-*GAL4*/*sima-RNAi* under hypoxia at 5% O_2_ (E). White arrows indicate clusters of satellite boutons. Scale bars represent 10 μm. (B, D, F) Bar graphs show percentages (mean ± SEM) of satellite boutons. Statistical significance was assayed by one-way ANOVA with Tukey's Multiple Comparison post-test (B) or Mann-Whitney test (D, F) with n.s., no significance; *, p <0.05; **, p < 0.01; ***, p < 0.001.

HIF-1α/Sima mediates the response to low oxygen supply [30, 31]. The protein levels of Sima are increased in wild-type *Drosophila* embryos subjected to hypoxia [32], leading to transcriptional activation of downstream target genes and the induction of tracheal branching [12]. We overexpressed Sima in tracheal cells, neurons, glia, or muscle cells by tissue-specific drivers to investigate which types of cells may play a role in modulating synaptic bouton formation. Overexpressing Sima in trachea caused embryonic lethality, preventing us from observing NMJ phenotypes. Larvae in which Sima was overexpressed in muscles, neurons, or glia could survive to the third instar stage, allowing us to examine the bouton phenotypes. We found that Sima overexpression in glia gave the highest number of satellite boutons (Fig 3A, and 11.9 ± 2.3%, n = 10 for *repo-GAL4/UAS-sima* in Fig 3B, v.s. 2.8 ± 1.0%, n = 10 for *repo-GAL4* in Fig 1D). However, Sima overexpression in neurons by *elav-GAL4* failed to induce satellite boutons (2.6 ± 0.8%, n = 8 in *elav-GAL4/UAS-sima* in Fig 3B v.s. 2.0 ± 0.7%, n = 10 for *elav-GAL4* in Fig 1D). Also, muscle expression of Sima also maintained a basal level of satellite boutons (1.7 ± 1.0%, n = 10 for *MHC-GAL4/UAS-sima* in Fig 3B v.s. 1.3 ± 0.8%, n = 10 for *MHC-GAL4* in Fig 1D). This result shows that the hypoxia-responding factor Sima is capable of inducing satellite bouton formation when overexpressed in glia.

The *Drosophila* PHD protein Fatiga (Fga) promotes prolyl hydroxylation and degradation of Sima in normaxia [33]. Consistently, glial knockdown by expressing *fga-RNAi* led to a significant increase of satellite boutons (S3A and S3B Fig, 8.3 ± 1.3%, n = 10 for *repo-GAL4/fga-RNAi*) from the control (4.3 ± 0.7%, n = 10 for *fga-RNAi*). Instead, tracheal knockdown by *btl-GAL4* (3.8 ± 0.7%, n = 9 for *btl-GAL4/fga-RNAi*) showed no apparent difference to the control. This result is consistent with that Sima upregulation in glia induces satellite bouton formation.

If glial Sima is the factor responding to hypoxia in the *trh*^*1*^*/trh*^*2*^ mutant, reducing Sima level in glia would suppress satellite bouton formation. Accordingly, we expressed the *sima-RNAi* transgene, which could reduce *sima* expression in both transcript and protein levels (S3C and S3D Fig), using *repo-GAL4* in the *trh*^*1*^*/trh*^*2*^ mutant. As our prediction, the satellite bouton phenotype was suppressed upon glial *sima* knockdown (3.6 ± 1.0%, n = 10 in *repo-GAL4/sima-RNAi trh*^*1*^*/trh*^*2*^), as compared to controls of the *trh*^*1*^*/trh*^*2*^ mutant carrying either the *UAS-sima-RNAi* transgene (9.2 ± 1.5%, n = 10 in *sima-RNAi trh*^*1*^*/trh*^*2*^) or the *repo-GAL4* driver (12.2 ± 2.2%, n = 10 in *repo-GAL4 trh*^*1*^*/trh*^*2*^) that displayed high percentages of satellite boutons (Fig 3C and 3D). We also tested whether low oxygen level-induced satellite bouton formation is mediated through Sima in glia. Satellite bouton phenotypes were detected in controls carrying either *UAS-sima-RNAi* (12.5 ± 2.7%, n = 11) or *repo-GAL4* (16.4 ± 2.2%, n = 10) when raised in 5% O_2_. However, almost no satellite boutons were detected in larvae carrying both *repo-GAL4* and *UAS-sima-RNAi* (0.45 ± 0.45%, n = 11 in *repo-GAL4/sima-RNAi*) when raised in the same condition (Fig 3E and 3F).

We also examined whether the Sima protein levels are changed in hypoxia or in the *trh* mutant. We found ubiquitous increases in the Sima levels in the wild-type control under the 5% O_2_ condition or in the *trh*^*1*^*/trh*^*2*^ mutant (S3E and S3F Fig). The increases could be identified in glial processes along the peripheral nerves and in different subtypes of glia. Thus, glial Sima could play the role to mediate hypoxia in the *trh*^*1*^*/trh*^*2*^ mutant and in the low O_2_ condition to modulate synaptic bouton formation.

### Wg signals mediate glial Sima activity to modulate bouton morphology

**Fig 4:**
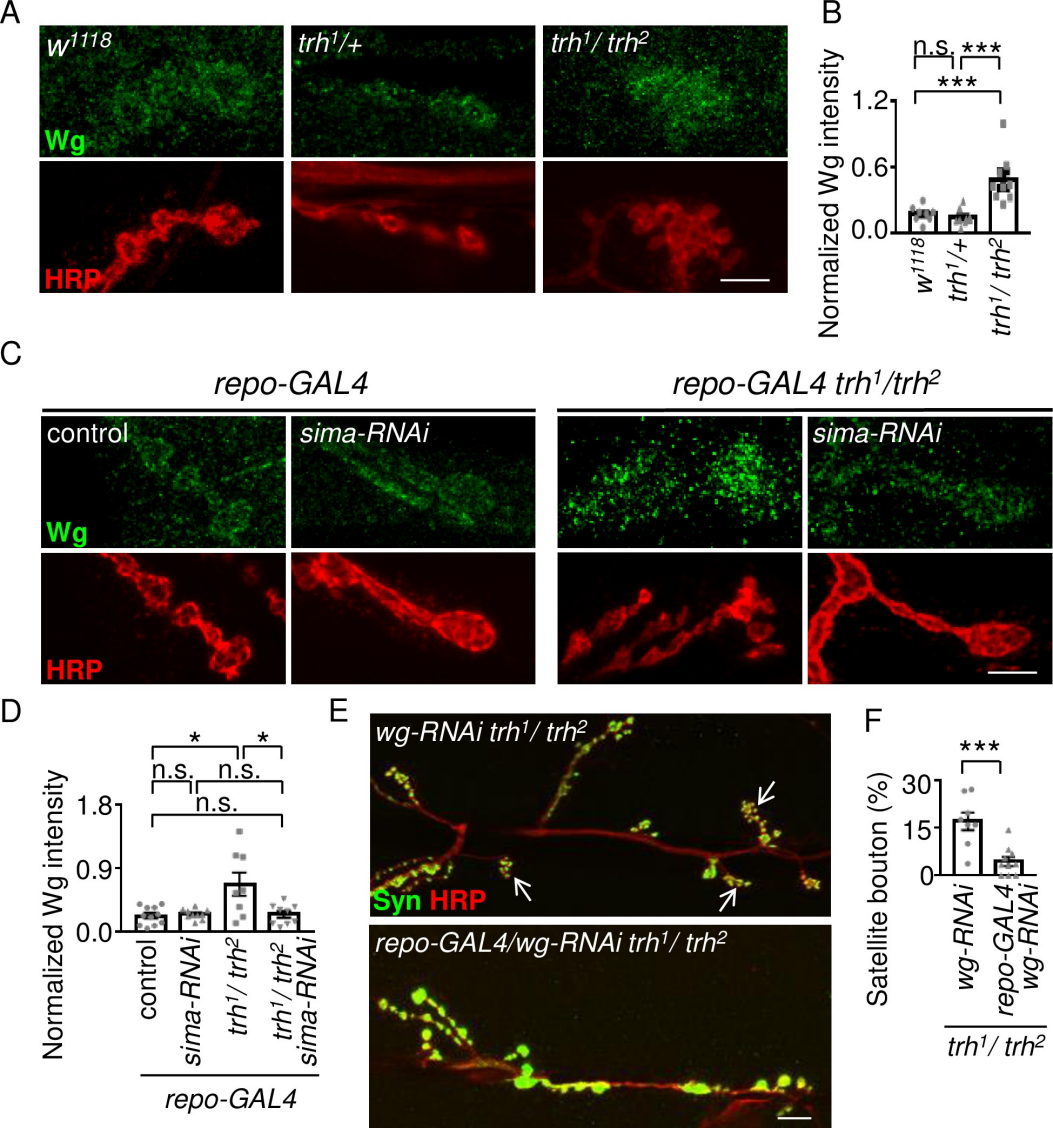
Wg induction from glia modulates synapse formation at *trh* NMJs. (A, C, E) Images showing NMJs of muscle 6/7 immunostained for Wg (green) and HRP (red) in *w*^*1118*^, *trh*^*1*^*/+*, and *trh*^*1*^*/trh*^*2*^ (A); *repo-GAL4*, *repo-GAL4*/*sima-RNAi*, *repo-GAL4 trh*^*1*^*/trh*^*2*^, and *repo-GAL4/sima-RNAi trh*^*1*^*/trh*^*2*^ (C); and for Syn (green) and HRP (red) in *wg-RNAi trh*^*1*^*/trh*^*2*^ and *repo-GAL4/wg-RNAi trh*^*1*^*/trh*^*2*^ (E). White arrows indicate clusters of satellite boutons. Scale bars represent 5 μm. (B, D, F) Bar graphs show averages (mean ± SEM) of normalized Wg to HRP intensities (B, D), and percentages (mean ± SEM) of satellite boutons (F). Statistical significance by Mann-Whitney test is shown (n.s., no significance; *, p < 0.05; ***, p < 0.001).

Next, we explored possible signals transduced from glia to neurons in response to hypoxia. The glia-secreted Wingless (Wg) signaling molecule regulates synaptic growth at *Drosophila* NMJs [34, 35]. Therefore, we examined whether Wg can be induced under hypoxia in the *trh*^*1*^*/trh*^*2*^ mutant. Wg signals were enriched around the synaptic boutons of wild-type NMJs (Fig 4A). Whereas the pattern of Wg signals at *trh*^*1*^*/+* NMJs was similar to that of *w*^*1118*^, we detected much higher levels of Wg signals at the *trh*^*1*^*/trh*^*2*^ NMJ (Fig 4A). Quantification of Wg immunofluorescence intensities normalized to co-stained HRP in *trh*^*1*^*/trh*^*2*^ (Wg/HRP: 0.49 ± 0.07, n = 9, Fig 4B) revealed ~3-fold increases relative to *w*^*1118*^ (0.18 ± 0.03, n = 8) and *trh*^*1*^*/+* (0.15 ± 0.02, n = 9). We then examined whether glial Sima is required for the enhanced Wg expression in the *trh*^*1*^*/trh*^*2*^ mutant (Fig 4C). The Wg level relative to the HRP level in the *trh*^*1*^*/trh*^*2*^ mutant carrying *repo-GAL4* (0.68 ± 0.16, n = 8 for *repo-GAL4 trh*^*1*^*/trh*^*2*^) was also about 3 folds to the *repo-GAL4* control (0.23 ± 0.04, n = 11, Fig 4D). When we reduced *sima* levels in the *trh*^*1*^*/trh*^*2*^ mutant by *repo-GAL4*-driven *UAS-sima-RNAi*, Wg signals were suppressed to a level equivalent to that in the *repo-GAL4* control (0.25 ± 0.05, n = 9 for *repo-GAL4/sima-RNAi trh*^*1*^*/trh*^*2*^). Interestingly, *sima-RNAi* knockdown in glia of the *repo-GAL4* control had no effect on the Wg level (0.25 ± 0.02, n = 10 for *repo-GAL4/sima-RNAi*), suggesting that Sima is induced in the *trh*^*1*^*/trh*^*2*^ mutant to upregulate Wg expression but has no role in basal Wg expression in the wild-type. Taken together, we suggest that glial Sima is required for Wg upregulation at the NMJs of the *trh*^*1*^*/trh*^*2*^ mutant.

If glia-secreted Wg is responsible for satellite bouton induction in the *trh*^*1*^*/trh*^*2*^ mutant, then glia-specific *wg* knockdown should phenocopy *sima* knockdown in glia to suppress satellite bouton formation. In control, satellite boutons were still prominent in the *trh*^*1*^*/trh*^*2*^ mutant bearing only *UAS-wg-RNAi* (Fig 4E and 4F, 17.0 ± 2.8%, n = 8 for *wg-RNAi trh*^*1*^*/trh*^*2*^). The satellite bouton phenotype was suppressed in the *trh*^*1*^*/trh*^*2*^ mutant bearing both *repo-GAL4* and *UAS-wg-RNAi* (4.4 ± 1.4%, n = 10 for *repo-GAL4/wg-RNAi trh*^*1*^*/trh*^*2*^). Also, overexpression of Sima by *repo-GAL4* induced satellite bouton formation (Fig 3B, 11.9 ± 2.3%, n = 10 for *repo-GAL4/UAS-sima*), which was suppressed by co-expressing the *wg-RNAi* transgene (Fig 3B, 2.3 ± 1.3%, n = 7 for *repo-GAL4/UAS-sima UAS-wg-RNAi*). Taken together, these results strongly suggest that glia-secreted Wg mediates Sima activity in promoting satellite bouton formation in the *trh* mutant.

**Fig 5:**
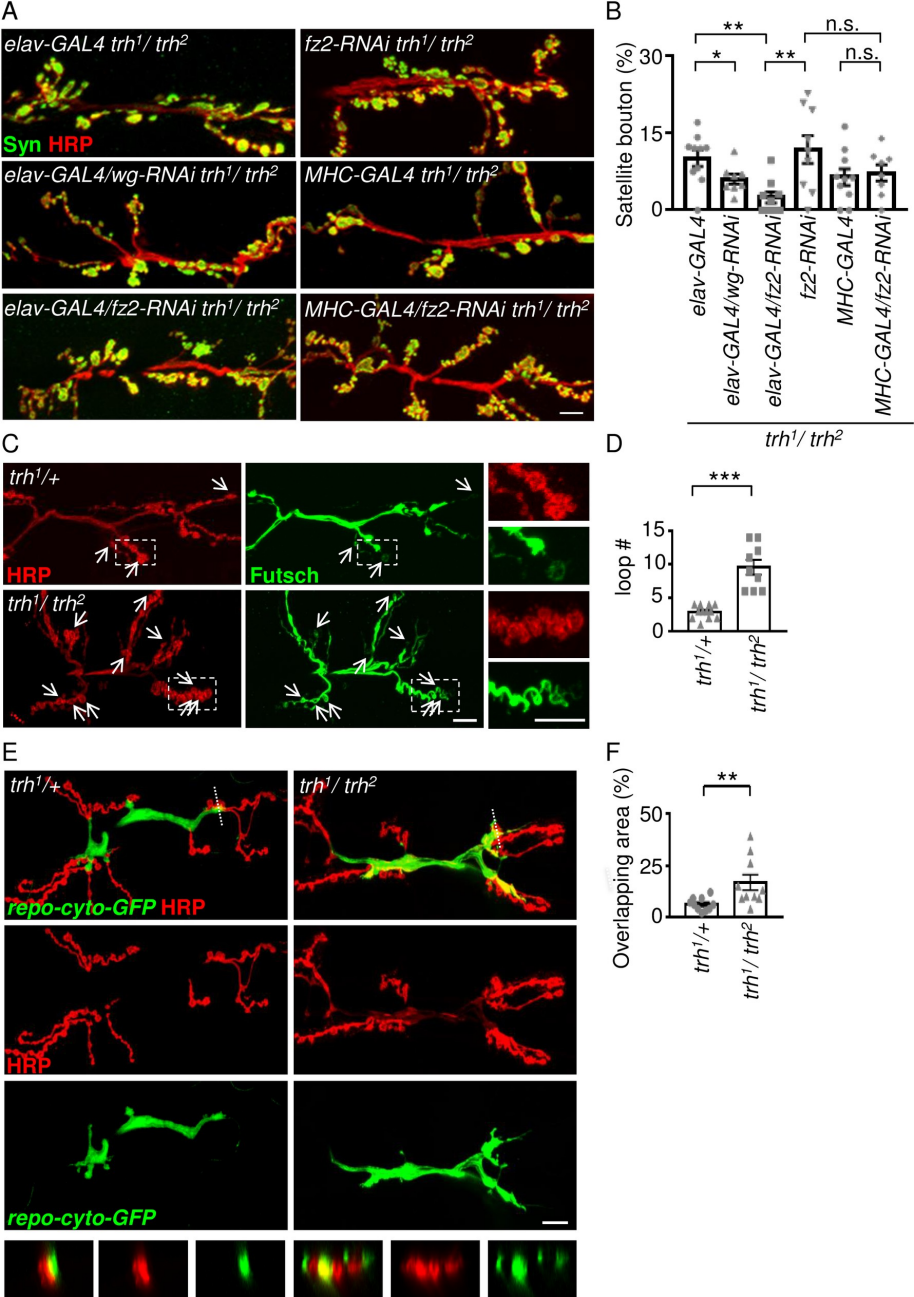
Presynaptic Wg signaling in modulating microtubule loops. (A) Images showing NMJ 6/7 immunostained for Syn (green) and HRP (red) in *elav-GAL4 trh*^*1*^*/trh*^*2*^, *elav-GAL4/wg-RNAi trh*^*1*^*/trh*^*2*^, *elav-GAL4/fz2-RNAi trh*^*1*^*/trh*^*2*^, *fz2-RNAi trh*^*1*^*/trh*^*2*^, *MHC-GAL4 trh*^*1*^*/trh*^*2*^, and *MHC-GAL4/fz2-RNAi trh*^*1*^*/trh*^*2*^. Scale bars represent 10 μm. (B) Bar graph shows averages (mean ± SEM) of percentages of satellite boutons. (C) Images showing NMJs of muscle 6/7 immunostained for HRP (red) and Futsch (green) in *trh*^*1*^*/+* and *trh*^*1*^*/ trh*^*2*^. White arrows indicate microtubule loops, and images within dotted squares are amplified in right panels. Scale bars represent 10 μm. (D) Bar graphs show average numbers (mean ± SEM) of Futsch loops. (E) Images of live tissues showing NMJs of muscle 6/7 carrying *repo-cyto-GFP* (green) labeled for HRP (red) in *trh*^*1*^/+ and *trh*^*1*^*/trh*^*2*^. Synaptic overlap by glia is shown as yellow color. Sections of overlapping boutons with dashed lines are shown in bottom panels. Scale bar represents 10 μm. (F) Bar graph shows percentages (mean ± SEM) of synaptic areas overlapping with glial processes. Statistical significance by Mann-Whitney test is shown (n.s., no significance; *, p < 0.05; **, p < 0.01; ***, p < 0.001).

As Wg signals are secreted from both glia and presynaptic neurons [34, 36], we found that reduction of Wg signals from neurons also suppressed satellite bouton formation in the *trh*^*1*^*/trh*^*2*^ mutant (Fig 5A and 5B, 10.1 ± 1.5%, n = 10 for *elav-GAL4 trh*^*1*^*/trh*^*2*^; 6.0 ± 1.0%, n = 8 for *elav-GAL4/wg-RNAi trh*^*1*^*/trh*^*2*^). Also, neuronal and glial but not muscle overexpression of Wg induced satellite bouton formation (S4A and S4B Fig). Glial and neuronal knockdown of Wg reduced total Wg levels at the *trh*^*1*^*/trh*^*2*^ NMJ, although the reduction by neuronal knockdown was not significant (S4C and S4D Fig). Thus, neuronal Wg also contributes to satellite bouton formation. However, only glial overexpression of Sima induced higher levels of Wg, but not neuronal or muscle overexpression of Sima (S4E and S4F Fig). Thus, while neuronal expression of Wg contributes to the overall level at NMJs in the *trh* mutant, Sima-induced Wg expression is likely glial-specific.

### Presynaptic Wg signaling modulates bouton morphology in the *trh* mutant

At larval NMJs, Wg signaling in pre-synaptic and post-synaptic sites through distinct signaling pathways [35–37]. However, the receptor Frizzled2 (Fz2) is involved in transducing the signaling pathway activities in both sites. We tested whether pre- or post-synaptic Wg signaling is involved in satellite bouton formation by *fz2* knockdown in the *trh* mutant. Neuronal expression of *fz2-RNAi* by *elav-GAL4* suppressed satellite bouton formation in the *trh*^*1*^*/trh*^*2*^ mutant (Fig 5A and 5B, 11.8 ± 2.7%, n = 9 for *fz2-RNAi trh*^*1*^*/trh*^*2*^; 10.1 ± 1.5%, n = 10 for *elav-GAL4 trh*^*1*^*/trh*^*2*^; and 2.5 ± 1.1%, n = 9 for *elav-GAL4/fz2-RNAi trh*^*1*^*/trh*^*2*^). In contrast, muscle expression of *fz2-RNAi* by *MHC-GAL4* had no significant difference to the controls (6.4 ± 1.6%, n = 10 for *MHC-GAL4 trh*^*1*^*/trh*^*2*^ and 7.3 ± 1.6%, n = 8 for *MHC-GAL4/fz2-RNAi trh*^*1*^*/trh*^*2*^). Thus, the elevated Wg level in the *trh* mutant is mainly transduced in presynaptic sites to modulate satellite bouton formation.

Inactivation of Wg signaling leads to a reduction of the more stabilized microtubule loops within synaptic boutons, which could be visualized by immunostaining for the microtubule-binding protein Futsch [35, 36]. We examined whether elevated Wg signaling alters microtubule loops in presynaptic boutons of the *trh*^*1*^*/trh*^*2*^ mutant by Futsch immunostaining and found significantly more Futsch-positive loops within the boutons of the *trh* mutant (Fig 5C and 5D, 2.92 ± 0.29, n = 12 for *trh*^*1*^*/+*; and 9.56 ± 1.11, n = 9 for *trh*^*1*^*/trh*^*2*^), supporting the elevation of presynaptic Wg signaling.

Glial processes invade synaptic boutons to match the growth of NMJs [38], which intrigued us to assess whether glia in the *trh*^*1*^*/trh*^*2*^ mutant exhibits morphological change. In a live fillet preparation for imaging NMJs, we found that glial processes labeled by GFP invaded the area of synaptic boutons in the *trh*^*1*^*/trh*^*2*^ mutant, whereas glial processes were relatively restrained from the bouton areas in the control (Fig 5E). Quantification of the glial process overlaying the synaptic bouton area revealed significantly greater area of overlap in the *trh*^*1*^*/trh*^*2*^ mutant relative to control (Fig 5F, 6.2 ± 1.0%, n = 10 for *trh*^*1*^*/+*; and 16.7 ± 3.7%, n = 10 for *trh*^*1*^*/trh*^*2*^). This increased extent of glial processes in the synaptic area may facilitate signal transduction from glia to synaptic boutons for structural reorganization. Taken together, these results suggest that Wg plays a prominent role in the *trh*^*1*^*/trh*^*2*^ mutant to transduce the hypoxia signal from glia to modify presynaptic bouton structure.

### Impaired crawling behavior in the *trh* mutant

**Fig 6:**
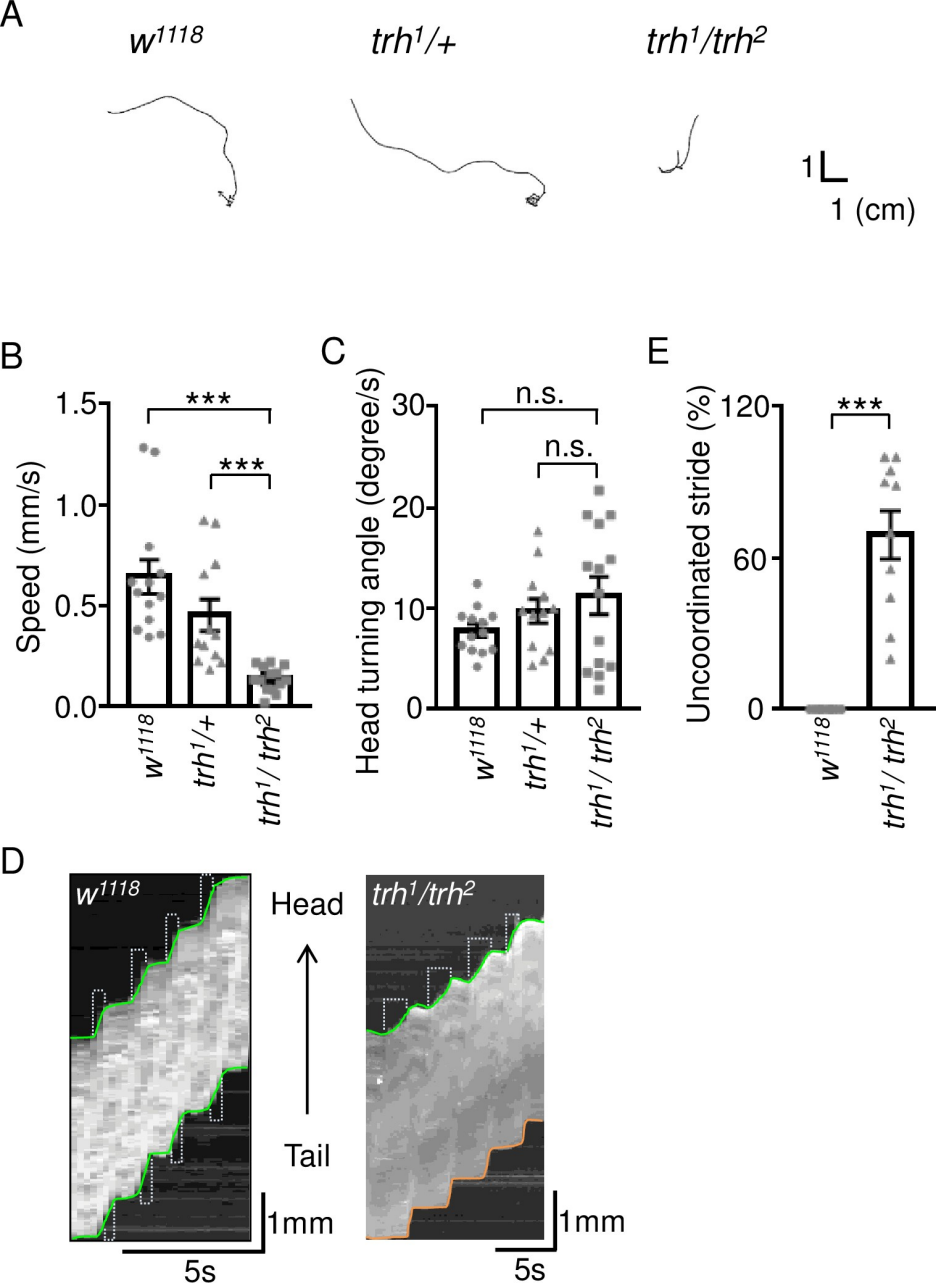
Impaired crawling behavior of the *trh* mutant. (A) Representative 5-minute crawling traces for *w*^*1118*^, *trh*^*1*^*/+*, and *trh*^*1*^*/trh*^*2*^. (B, C) Bar graphs show (B) average speeds (mean ± SEM in mm/s), and (C) average rotational speeds (mean ± SEM in degree/s). (D) Kymographs show four forward strides from tail to head (shown by arrow) for *w*^*1118*^ and *trh*^*1*^*/trh*^*2*^. The green lines show smooth transitions between successive strides, whereas orange lines show abrupt transitions. Scale bars for 5s and 1mm are shown. (E) Bar graph shows uncoordinated stride percentages (mean ± SEM). Statistical significance by Mann-Whitney test is shown (n.s., no significance; ***, p < 0.001).

Given the evident morphological changes at *trh*^*1*^*/trh*^*2*^ NMJs, we wondered if locomotion is affected in mutant larvae. We observed larvae crawling under free-movement conditions and found that wild-type control and *trh*^*1*^*/+* heterozygous larvae presented smooth crawling paths (Fig 6A), with an average speed of 0.64 ± 0.09 mm/s (n = 13) in *w*^*1118*^ and 0.45 ± 0.08 mm/s (n = 12) in *trh*^*1*^*/+* (Fig 6B). However, the *trh*^*1*^*/trh*^*2*^ larvae had much shorter paths and a slower speed of 0.14 ± 0.02 mm/s (n = 14). The head turning angle of the *trh*^*1*^*/trh*^*2*^ mutant was comparable to both controls, not contributing to the slow movement (Fig 6C, 7.8 ± 0.6 degree/s, n = 13 for *w*^*1118*^; 9.7 ± 1.2 degree/s, n = 12 for *trh*^*1*^*/+*; and 11.3 ± 1.9 degree/s, n = 14 for *trh*^*1*^*/trh*^*2*^). Larval crawling is a rhythmic behavior involving a series of periodic strides (S1 Movie) [39]. We noticed uncoordinated crawling in the *trh*^*1*^*/trh*^*2*^ larvae, with their posterior body segments failing to follow the rhythmic movement (S2 Movie). We recorded larval forward crawling and constructed kymographs to represent the stride cycle. In wild-type larvae, normal and consistent periodic strides were apparent with regular head and tail displacements (Fig 6D, left panel). Similar to the wild-type, head movements of *trh*^*1*^*/trh*^*2*^ larvae were smooth and periodic, albeit slower. However, tail movements of *trh*^*1*^*/trh*^*2*^ larvae were abrupt (Fig 6D, right panel). While wild-type larvae crawled completely normal (n = 10), 69.2 ± 9.6% (n = 10) of the strides of *trh*^*1*^*/trh*^*2*^ larvae were uncoordinated (Fig 6E), which might contribute to the slower crawling of the *trh*^*1*^*/trh*^*2*^ mutant.

**Fig 7:**
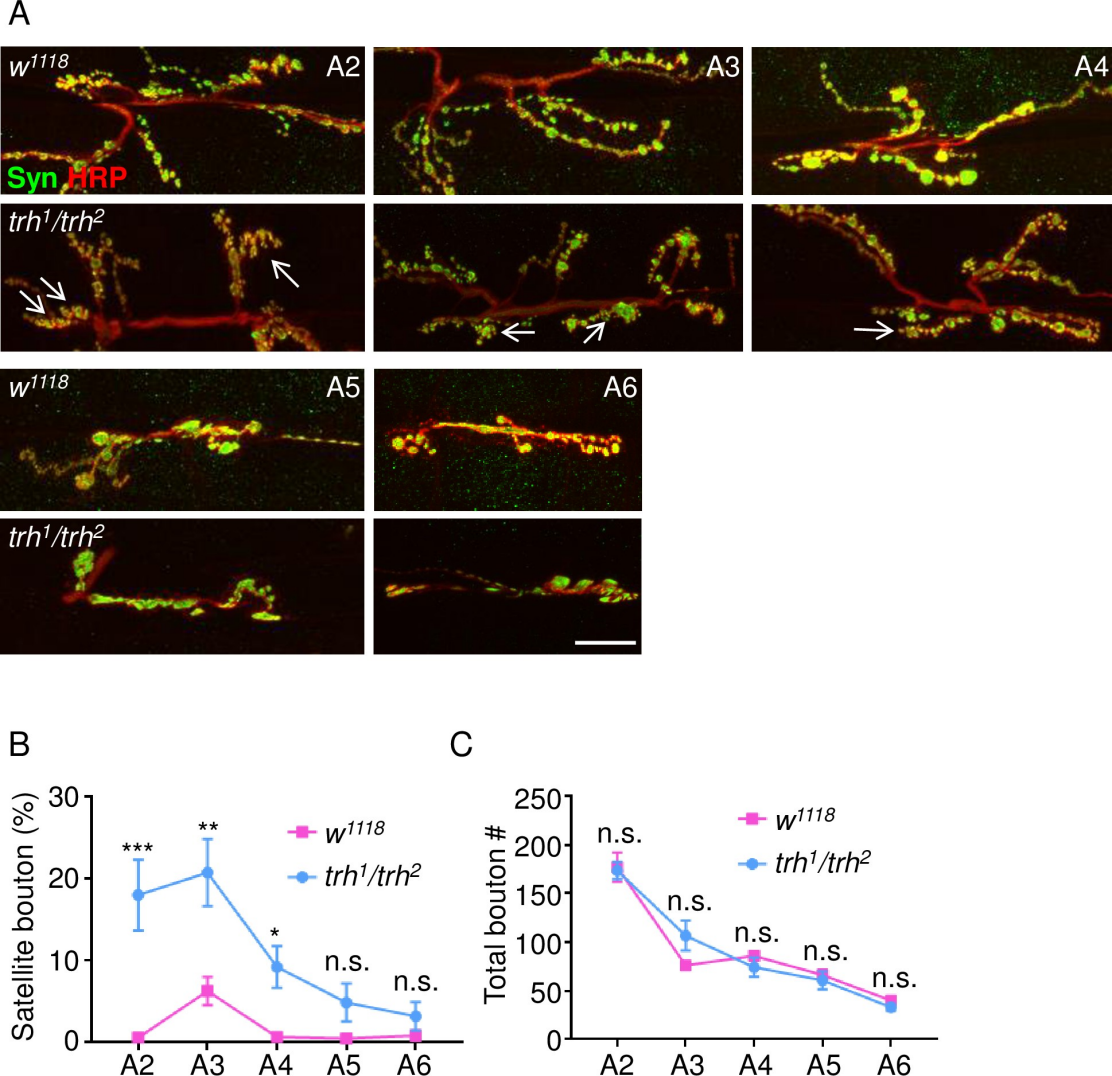
Selective satellite bouton formation at anterior segments of *trh* NMJs. (A) Images show NMJ 6/7 from segments A2-A6 immunostained for Syn (green) and HRP (red) in *w*^*1118*^ and *trh*^*1*^*/ trh*^*2*^. White arrows indicate clusters of satellite boutons. Scale bar represents 10 μm. (B, C) Line graphs show average percentages (mean ± SEM) of satellite boutons (B) and total bouton numbers (C) for *w*^*1118*^ (magenta lines) and *trh*^*1*^*/ trh*^*2*^ (blue lines). Statistical significance by Mann-Whitney test is shown (n.s., no significance; *, p < 0.05; **, p < 0.01; ***, p < 0.001).

This uncoordinated stride cycle prompted us to examine the bouton morphology in A2-A6 segments of the *trh*^*1*^*/trh*^*2*^ larvae (Fig 7A and 7B). Strikingly, large numbers of satellite boutons were detected in segments A2 (18.0 ± 4.3%, n = 10) and A3 (20.7 ± 4.1%, n = 10) and an intermediate level of satellite boutons was detected in segment A4 (9.2 ± 2.6%, n = 9). The numbers of satellite boutons were low in more posterior segments A5 (4.9 ± 2.3%, n = 10) and A6 (3.2 ± 1.7%, n = 9). In wild-type larvae, all segments had relatively normal bouton morphology except the A3 segment (0.6 ± 0.4%, n = 10 in A2, 6.3 ± 1.7%, n = 10 in A3, 0.7 ± 0.4%, n = 10 in A4, 0.5 ± 0.5%, n = 10 in A5, and 0.9 ± 0.6%, n = 9 in A6). While high numbers of satellite boutons appeared in the *trh* mutant, the total numbers of boutons in individual segments were comparable between wild-type and *trh*^*1*^*/trh*^*2*^ (Fig 7C, *w*^*1118*^: A2, 176.6 ± 15.0, n = 10; A3, 76.1 ± 4.8, n = 9; A4, 85.8 ± 4.1, n = 10; A5, 66.1 ± 5.4, n = 10; A6, 40.0 ± 3.7, n = 9; *trh*^*1*^*/trh*^*2*^: A2, 173.2 ± 8.8, n = 10; A3, 106.6 ± 15.3, n = 10; A4, 74.1 ± 9.9, n = 9; A5, 60.7 ± 9.1, n = 10; A6, 33.1 ± 3.6, n = 9). Therefore, these analyses suggest that satellite boutons are prone to appear in more anterior than posterior segments.

We further examined whether glial Sima and Wg have any role on modifying bouton morphology in the posterior A6 segment of the *trh*^*1*^*/trh*^*2*^ larvae. With the satellite boutons at a basal level in the A6 segment of *trh*^*1*^*/trh*^*2*^ (Fig 7B), we tested whether overexpression of Sima or Wg could induce satellite boutons in the *trh*^*1*^*/trh*^*2*^ mutant. Overexpression of Sima by *repo-GAL4* in *trh*^*1*^*/trh*^*2*^ induced some satellite boutons (S5A and S5B Fig, 6.8 ± 2.3%, n = 10), which showed no significant difference to the *trh*^*1*^*/trh*^*2*^ mutant carrying *repo-GAL4* (2.8 ± 1.5%, n = 8). Overexpression of Wg by *repo-GAL4* in *trh*^*1*^*/trh*^*2*^ displayed a basal level of satellite boutons (2.6 ± 1.5%, n = 9). Also, glial *wg-RNAi* knockdown suppressed satellite bouton formation in the A3 segment of *trh*^*1*^*/trh*^*2*^ (Fig 4F), but had no effect on the morphological phenotype in the A6 segment (S5C and S5D Fig). Thus, the analysis of these data suggests that Wg and Sima might have relatively specific roles to induce satellite bouton formation in the anterior A3 segment.

**Fig 8:**
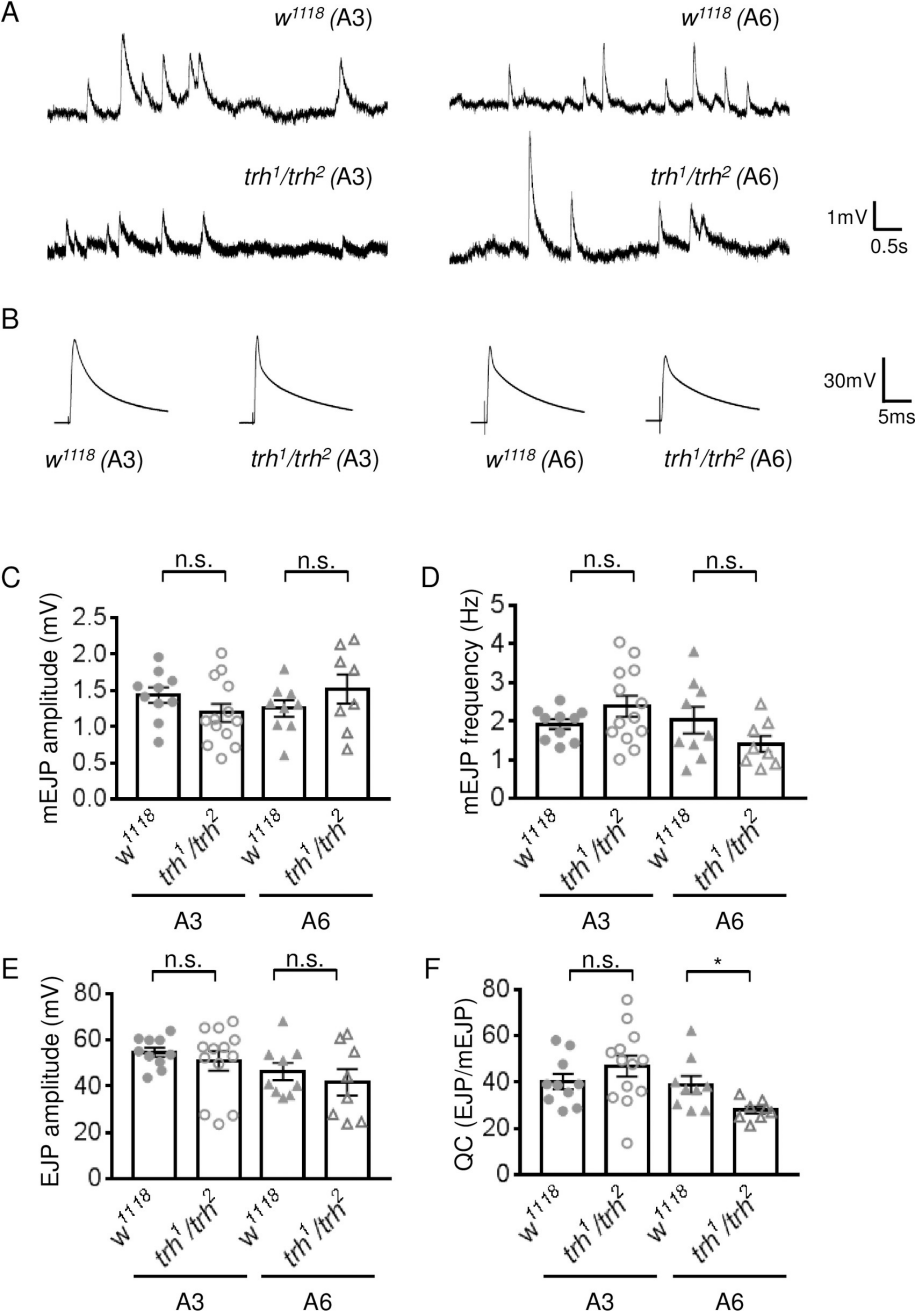
Synaptic transmission at *trh* NMJs. (A, B) Representative traces of mEJPs (A) and EJPs (B) recorded from NMJs of muscle 6 of segment A3 (left panels) and A6 (right panels) for *w*^*1118*^ and *trh*^*1*^*/trh*^*2*^. The scales for both mEJP and EJP traces are depicted at right. (C-F) Bar graphs show averages (mean ± SEM) for (C) mEJP amplitudes, (D) mEJP frequencies, (E) EJP amplitudes and (F) quantal contents (QC) calculated as EJP/mEJP. Statistical significance by Mann-Whitney test is shown (n.s., no significance and *, p < 0.05).

Given the satellite bouton phenotype in the *trh*^*1*^*/trh*^*2*^ larvae, we assessed basal synaptic transmission properties, firstly at muscle 6 of the A3 segment. The amplitude of spontaneous release, or the miniature evoked junctional potential (mEJP), was slightly but non-significantly reduced (Fig 8A), from 1.4 ± 0.1 mV (n = 10) in *w*^*1118*^ to 1.2 ± 0.1 mV (n = 13) in *trh*^*1*^*/trh*^*2*^ (Fig 8C). Comparable frequencies were detected between *w*^*1118*^ (1.9 ± 0.1 Hz, n = 10) and *trh*^*1*^*/trh*^*2*^ (2.4 ± 0.3 Hz, n = 13)(Fig 8D). The amplitudes of EJP were also comparable between *w*^*1118*^ (54.6 ± 2.1 mV, n = 10) and *trh*^*1*^*/trh*^*2*^ (50.9 ± 4.2 mV, n = 13)(Fig 8B and 8E). The quantal content, calculated by dividing the EJP amplitude with that of mEJP, was slightly but non-significantly increased, from 40.2 ± 3.3 (n = 10) in *w*^*1118*^ to 46.9 ± 4.5 (n = 13) in *trh*^*1*^*/trh*^*2*^ (Fig 8F). We then evaluated the synaptic transmission properties of muscle 6 for the A6 segment. Between the *w*^*1118*^ control and the *trh*^*1*^*/trh*^*2*^ mutant, the mEJP amplitudes (1.2 ± 0.1, n = 9 v.s. 1.5 ± 0.2 mV, n = 8), the mEJP frequencies (2.0 ± 0.3 Hz, n = 9, v.s. 1.4 ± 0.2 Hz, n = 8), and the EJP amplitudes (46.2 ± 3.7 mV, n = 9 v.s. 41.6 ± 5.7 mV, n = 8) remained similar without significant difference (Fig 8A–8E). However, the slight increase in mEJP and the slight decrease in EJP in *trh*^*1*^*/trh*^*2*^ lead to a significant reduction in the quantal content, from 38.9 ± 3.7 (n = 9) in *w*^*1118*^ to 27.9 ± 1.5 (n = 8) in *trh*^*1*^*/trh*^*2*^ (Fig 8F). Thus, the impaired synaptic activity of the A6 segment in the mutant larvae may underlie the defective stride cycles of the posterior segments.

## Discussion

Here, we demonstrate that Trh, a member of the NPAS protein family, non-cell autonomously regulates synaptic bouton formation at NMJs through a hypoxic response from glia. We observed small-sized and clustered satellite boutons at the NMJs of the *trh* mutant larvae or larvae reared at low oxygen levels. The abnormal bouton morphology at the *trh* NMJs could be suppressed by reducing the level of the hypoxia-inducible factor Sima in glia. We further show that Sima enhanced the Wg signal from glia to cause satellite bouton formation. Although normal synaptic transmission was detected at NMJs located in an anterior segment of larvae bearing satellite boutons, reduced synaptic transmission was found in a posterior segment lacking satellite boutons of the *trh* mutant, suggesting that glia-induced satellite bouton formation might be a homeostatic response in restoring normal synaptic transmission. Imbalanced synaptic activities at the anterior and posterior NMJs of the *trh* mutant might contribute to the uncoordinated stride cycles detected in the *trh* mutant, slowing larval crawl speed (S6 Fig). Thus, we provide a model for studying the glial responses that modulate synaptic bouton reorganization and activities during hypoxia.

### Glia play a critical role in satellite bouton formation in the *trh* mutant

Our results suggest that Trh has a late developmental role in tracheal morphogenesis, in addition to its well-characterized role in early tracheal cell fate specification [23, 24]. We observed defective tracheal structure in the *trh* mutant (S2A Fig), which may result in hypoxic conditions inside the larval body. The increase in terminal branch number (S2A and S2B Fig) may be a response to oxygen supply deficiency [12]. Moreover, the increases in Sima protein levels and *ODD-GFP* reporter expression indicate reduced internal oxygen levels (Fig 2A and 2B and S3E and S3F Fig). Finally, the satellite bouton phenotype in the *trh* mutant was recapitulated by rearing larvae under hypoxia, and it was suppressed by rearing larvae under hyperoxia. Taken together, these observations suggest that cells in the *trh* mutant sense low oxygen levels caused by the defective tracheal system and respond by elevating Sima protein levels. It is not clear how profound this effect is for other types of larval cells. Based on our ODD-GFP and Sima immunostaining patterns (Fig 2A and 2B and S3E and S3F Fig), many types of cells are likely to be affected [40].

We suggest that glia is the major cell type mediating satellite bouton formation in the *trh* mutant under hypoxia. While Sima was increased ubiquitously, manipulating the levels of Sima or Ftg, the negative regulator of Sima, in glia modulates satellite bouton formation (Fig 3 and S3A and S3B Fig). Elevated Sima levels induce tracheal sprouting in tracheal cells, as well as protrusions in non-tracheal cells [12]. Interestingly, we also observed protrusion of glial processes into synaptic area in the *trh* mutant, indicative of a glial response (Fig 5E and 5F). Several types of cells in *Drosophila* have been shown to respond to hypoxia [32, 41]. For instance, under hypoxia, elevated Sima levels induce the expression of Breathless (Btl, the FGF receptor) in tracheal cells that branch out seeking cells that express Branchless (Bnl)/FGF, with this latter process also being partially dependent on Sima [12, 13]. In an alternative pathway, atypical soluble guanylyl cyclases can mediate graded and immediate hypoxia responses mainly in neurons [42, 43]. *Drosophila* glia have not been reported to sense and respond to hypoxia, but mammalian astrocytes in the central nervous system have been shown to be involved in these processes. In a mouse model for middle cerebral artery occlusion, astrocyte activation was shown to play a crucial role in ischemic tolerance, which is mediated through P2X7 receptor-activated HIF-1α upregulation [18]. Under physiological hypoxia, reduced mitochondrial respiration leads to the release of intracellular calcium and exocytosis of ATP-containing vesicles, thereby signaling the brainstem to modulate animal breathing [44]. Our results reveal a role for *Drosophila* larval glia in sensing hypoxia via the conventional HIF-1α/Sima pathway. We also demonstrate that under hypoxia, glia modulate the formation of synaptic boutons (Fig 3E and 3F). These results clearly place the glia-modulated morphology of synaptic boutons in the context of hypoxia responses.

### Sima elevates Wg signal expression in glia in satellite bouton formation

Our study further establishes that in response to hypoxia, Wg is a glial signal that modulates synaptic bouton formation. Two sources of Wg, presynaptic motor neurons and glia, are involved in synaptic growth and remodeling [34, 36]. Our results suggest that Sima upregulates the level of Wg secreted from glia to modulate synapse formation in the *trh* mutant or in control larvae grown under hypoxia. In hypoxic macrophages, HIF-1α ediates the induction of Wnt11, which is a mammalian homolog of Wg [45]. It is likely *wg* is a direct target of Sima in *Drosophila*. We found the HIF1-α binding motif (CGTG) at the -269 nucleotide sequence in the *wg* promoter. Also, direct binding of Sima to this consensus site was reported in a systematic ChIP-seq experiment [46]. We further show that the level of Wg is controlled by glial Sima in the *trh* mutant (Fig 4C and 4D) and that Wg mediates Sima-induced satellite bouton formation at *trh* NMJs (Fig 3B). Also, glial overexpression of Sima upregulates Wg levels at NMJs (S4E and S4F Fig). Wg is also expressed from presynapses [35]. Thus, neuronal *wg* knockdown partially suppressed satellite bouton phenotypes in the *trh* mutant (Fig 5A and 5B), while neuronal wg overexpression in wild-type induced the phenotypes (S4A and S4B Fig). These results are consistent with the idea that presynaptic Wg contributes to the overall pool of Wg at NMJs of the *trh* mutant. As a secreted morphogen, Wg functions in both pre-synaptic and post-synaptic sites [35–37]. At presynaptic terminals, the canonical Wg pathway induces microtubule loop formation to regulate synaptogenesis. We also detected an increase in microtubule loops in the *trh* mutant (Fig 5C and 5D), consistent with a role for Wg signaling in modulating synaptic reorganization. Postsynaptic Wg signaling leads to subsynaptic reticulum differentiation [35], which was not apparent in the *trh* mutant (S1D Fig), suggesting that Wg might be a component of the complex hypoxia response that induces synaptic bouton reorganization. Brief exposure to hypoxia induces immature spines and impaired synaptic function in hippocampal neurons [17]. The morphological change to satellite boutons at the A3 segment of the *trh* NMJs was not accompanied by altered synaptic transmission (Fig 8), which may be compensated during long-term hypoxia.

The satellite boutons, also named as bunch boutons, have been described in *spastin* mutants [47, 48]. As an AAA ATPase, Spastin severs microtubules to facilitate transport to distal axon segments [49]. Accordingly, the *spastin* mutant also exhibits a lack of microtubules at terminal boutons [48]. In contrast, the *trh* mutant presented an increase of the more stabilized microtubule loops (Fig 5C and 5D). Microtubule loops have been linked to synaptic bouton stabilization, and an excess of microtubule loops has been associated with increased synaptic bouton formation [50, 51]. The altered morphology of satellite boutons may be part of the structural changes necessary to maintain normal synaptic transmission under hypoxia. The *trh* and *spastin* mutants also exhibit differences in synaptic function, with loss of *spastin* function slightly enhancing spontaneous synaptic transmission release but reducing evoked synaptic transmission [48]. Thus, although the morphology of synaptic boutons at *trh* NMJs resembles that of *spastin* mutants, satellite boutons at *trh* NMJs retain synaptic functions, unlike the impaired synaptic transmission of *spastin* mutant boutons.

### Differential morphological and physiological changes of anterior and posterior segments in the *trh* NMJs

The size of NMJs in muscles 6/7 decreases from the anterior to posterior segments, which could represent a coupling with muscle growth [52, 53], thereby maintaining similar electrophysiological efficacy at anterior and posterior NMJs (Fig 8). Interestingly, our findings show that synaptic responses in the *trh* mutant differ, with satellite boutons only appearing in anterior segments (Fig 7A and 7B). Furthermore, synaptic transmission at *trh* NMJs remained normal in the anterior A3 segment but was impaired in the posterior A6 segment (Fig 8). These observations are consistent with the idea that satellite bouton formation is a part of a homeostatic response to restore synaptic activity. Why synapses are not reorganized in the posterior segments remains elusive. We failed to detect an upregulation of Wg in the A6 segment (S4C and S4D Fig), and glial Sima overexpression even in the A6 segment of the *trh* mutant failed to increase satellite boutons significantly (S5A and S5B Fig). Thus, the upregulation of Wg by Sima may be segment-dependent, which awaits further study. Motor neurons in the ventral nerve cord project much longer axons to muscles in posterior segments compared to anterior ones. It has been shown that axonal transport to posterior segments is more vulnerable to inefficient transport conditions. For example, mutation of long-chain Acyl-CoA synthetase impairs the balance between anterograde and retrograde transport, causing distally-biased axonal aggregations and affecting the growth and functioning of synapses [54]. It is possible that glia-derived Wg signals may not be efficiently transported to posterior segments during hypoxia. This polar difference in synaptic activity and bouton morphology may contribute to the uncoordinated movements of the *trh* mutant larvae. Alternatively, defective locomotion in posterior segments of the *trh* mutant is independent of the glial modulation of bouton morphological changes. Larval forward locomotion, propelled by peristaltic contraction, is controlled by different circuits targeting anterior and posterior segments. The GABAergic SEZ-LN1 neurons specifically control posterior A6 and A7 segmental muscle contraction by inhibiting A27h premotor neurons, which promotes longitudinal muscle contraction during larval forward crawling [55]. Specific alteration of the circuit in the posterior segments may lead to the locomotion defect in the *trh* mutant.

## Materials and methods

### Fly stocks

All flies were reared at 25°C. *w*^*1118*^ was used as wild-type control and to backcross with *trh*^*1*^ or *trh*^*2*^. The sources of fly strains are as follows: *trh*^*2*^, *elav-GAL4*, *MHC-GAL4*, *repo-GAL4*, *UAS-trh*, *UAS-sima*, *UAS-sima-RNAi*, *Nrv2-GAL4*, and *His2Av-mRFP* were obtained from Bloomington Drosophila Stock Center (BDSC); *trh*^*1*^, *NP6293-GAL4*, and *UAS-wg-RNAi* from Kyoto Stock Center; and *UAS-trh-RNAi*, *UAS-fga-RNAi*, and *UAS-fz2-RNAi* from Vienna Drosophila Resource Center (VDRC). Also used were *btl-GAL4*[56], *moody-GAL4* [57], *alrm-GAL4*[58], *UAS-wg*, and *GFP-ODD* [29]. The *repo-cyto-GFP* line was generated with the sequence for cytoplasmic GFP under the control of the 4.3 kb *repo* promoter, which recapitulates the full *repo* expression pattern.

### Hypoxia or hyperoxia rearing conditions

Larvae in a food vial were transferred at 1 day after egg laying (AEL) to a ProOx (model 110, BioSpherix, Lacona, NY) oxygen-controlled chamber. Oxygen or nitrogen was infused into the chamber to a desired concentration (5% or 50%), which was maintained until assay.

### Immunostaining

The NMJ phenotypes were analyzed as previously described [59]. For live tissue preparation to detect *repo-cyto-GFP* expression, non-fixed larvae were dissected and the larval fillets were incubated with anti-horseradish peroxidase (HRP, 1:10) in phosphate buffered saline (PBS) for 10 minutes. Primary antibodies used were against Synapsin (3C11, mouse, 1:100; Developmental Studies Hybridoma Bank, DSHB), HRP-Cy5 (rabbit, 1:100; Jackson ImmunoResearch), Dlg (mouse, 1:100, DSHB), GluRIIA (mouse, 1:100, DSHB), dPAK (rabbit, 1:1000), GluRIII (rabbit, 1:1000), Brp (nc82, mouse, 1:100, DHSB), Sima (guinea pig, 1:1000)[60], Repo (mouse, 1:1000, DSHB), Wg (4D4, mouse, 1:10, DSHB), and Futsch (22C10, mouse, 1:100, DSHB). Secondary antibodies used were anti-rabbit or -mouse 488, Cy3, or Cy5 (1:1000, Jackson ImmunoResearch). For Wg immunostaining, larval preparations of different genotypes were marked and immunostaining was performed in the same test tube.

### Image acquisition and processing

NMJs in muscle 6/7 of A3 segments (or A2-A6 in Fig 7 and A6 in S4 and S5 Figs) of wandering third-instar larvae were analyzed. Confocal images were acquired via LSM510 confocal microscopy (Carl Zeiss) using 40x water, 40x water immersion (for live tissue in Fig 5E), or 100x oil objectives. All presented images are projections of confocal z-stacks. Numbers of satellite boutons, total boutons, and microtubule loops were counted manually. The percentage of satellite boutons was calculated as the number of satellite boutons divided by the total boutons (satellite + normal ones) for each NMJ, and the average percentage is calculated from about 6–13 NMJs for each genotype. The immunofluorescence intensities of Wg and HRP were analyzed by ImageJ. HRP-positive regions were chosen to measure mean intensities of Wg and HRP. After subtracting the intensity with the background one, the ratio of Wg levels to HRP levels was presented as the normalized Wg intensity. The overlapping area of GFP and HRP projections was chosen by the “AND” operator in ImageJ, which was divided by HRP area for the percentage. Each dot in the bar graph represents the data from a single NMJ of a larva, and 6–13 NMJs from 2–5 independent experiments were pooled for quantification. Embryos were acquired by means of LSM510 confocal microscopy (Carl Zeiss) using a 20x objective, and were analyzed as previously described [29]. For Fig 2B, each dot in the bar graph represents data from a single embryo in which fluorescence was measured in at least 35 cells.

### Electrophysiological recordings

Basal transmission properties were analyzed at NMJs of muscle 6/7 in specified segments of wandering third-instar larvae as previously described [61], with some modifications. The larval body wall was dissected in cold calcium-free HL3 solution and recorded in HL3 solution containing 0.4 mM CaCl_2_ at room temperature.

### Crawling behavior

Mid third instar larvae (feeding stage) were placed on black agar plates (2% agar with black food coloring in 25 × 20 cm^2^ dishes) at room temperature for filming. Video recording by a Sony Xperia Z1 camera started after 1 min habituation and lasted for 5 min, and it was analyzed using Ctrax software [62]. The (x, y) positions were used to calculate the crawling distance between two successive frames, and crawling speed was derived by dividing total distance travelled by time. The change in angle of larvae between two frames was divided by time to represent rotational angles. The forward crawling assay was a modification of a previous study [39]. Larvae were transferred into a tunnel (~1 mm width) made in 2% black agar. Specimens were video-recorded for 3–10 minutes using a Leica S8 APO microscope. Kymographs were constructed using the MultipleKymograph plug-in for ImageJ (NIH). Only forward crawling was counted, and 7–10 steps for each of ten larvae were analyzed for each genotype.

### Statistics

Statistic data were analyzed by Mann-Whitney test or one-way ANOVA with Tukey's Multiple Comparison post-test, and shown by scatter plots with bar using GraphPad Prism.

## Supporting information

S1 Fig Presynaptic and postsynaptic structures in *trh* mutant. (A) Bar graphs show averages of muscle areas (*w*^*1118*^, 61.2 ± 1.6 x10^3^ μm^2^, n = 12; *trh*^*1*^*/+*, 70.0 ± 2.8 x10^3^ μm^2^, n = 11; and *trh*^*1*^*/trh*^*2*^, 60.5 ± 4.9 x10^3^ μm^2^, n = 9). (B, C, D) Images showing NMJs of muscle 6/7 immunostained for Brp, GluRIII, and HRP (B), GluRIIA, dPAK, and HRP (C), and Dlg and HRP (D) in *w*^*1118*^, *trh*^*1*^*/+*, and *trh*^*1*^*/trh*^*2*^. Scale bars are 10 μm. Statistical significance in (A) was assayed by Mann-Whitney test is shown (n.s., no significance).
(TIF)

S2 Fig Tracheal defects in the *trh* mutant. (A) A bright-field view of tracheal dorsal branches of *trh*^*1*^*/+* (left top panel) and *trh*^*1*^*/trh*^*2*^ (right top panel), and tracheal dorsal trunks of *trh*^*1*^*/trh*^*2*^ (bottom panels). Numbers denote terminal branches, and arrows indicate a tracheal break (bottom left) and a tracheal tangle (bottom right). Scale bar represents 50 μm. (B) Bar graph shows averages of dorsal terminal branches. (C) Bar graph shows averages of the GFP immunofluorescence intensity (*w*^*1118*^ in 21% O_2_, 20.3 ± 7.5, n = 6; *w*^*1118*^ in 5% O_2_, 79.3 ± 18.0, n = 6; *trh*^*1*^*/+* in 21% O_2_, 13.3 ± 3.6, n = 6; *trh*^*1*^*/trh*^*2*^ at 21% O_2_, 108.2 ± 14.7, n = 6). (D) Bar graph shows averages of RFP immunofluorescence intensity (*w*^*1118*^ in 21% O_2_, 102.0 ± 15.5, n = 6; *w*^*1118*^ at 5% O_2_, 109.6 ± 13.1, n = 6; *trh*^*1*^*/+* at 21% O_2_, 143.0 ± 15.7, n = 6; *trh*^*1*^*/trh*^*2*^ at 21% O_2_, 151.5 ± 16.8, n = 6). Statistical significance by Mann-Whitney test is shown (n.s., no significance; *, p < 0.05; **, p < 0.01; ***, p < 0.001).
(TIF)

S3 Fig Fga in satellite bouton formation, efficiency of *sima-RNAi* knockdown and Sima upregulation in hypoxia and the *trh* mutant. (A) Images showing NMJs from muscle 6/7 immunostained for Syn (green) and HRP (red) in *fga-RNAi*, and *repo-GAL4/fga-RNAi* and *btl-GAL4/fga-RNAi*. (B) Bar graph shows percentages (mean ± SEM) of satellite boutons. Statistical significance was assayed by Mann-Whitney test (n.s., no significance; *, p <0.05). (C, D) RT-PCR for *sima* mRNA (C) or Western blot for Sima protein (D) expressions in *da-GAL4* (control) or *da-GAL4/sima-RNAi*. The controls *Rpl19* mRNA (C) and GAPDH protein (D) are comparable in both genotypes. (E) Images showing central nerve cord (left 3 panels) and peripheral nerve (right 2 panels) immunostained for Sima (green) and Repo (red) for *w*^*1118*^ in 21% O_2_, *w*^*1118*^ in 5% O_2_ for 4hrs, and *trh*^*1*^*/trh*^*2*^ in 21% O_2._ Arrows indicate nuclei of glia with high Sima protein levels. Enlarged images of a nucleus in each condition are shown in left bottom site. (F) Images showing Sima (green) localization in cells of His2Av-mRFP-expressing subperineurial (*moody-GAL4*), wrapping (*Nrv2-GAL4*), astrocyte-like (*alrm-GAL4*) and perineurial (*NP6293-GAL4*) glia in 5% O_2_ for 4hrs. Lateral views of the Sima-enriched subtype glia are shown in the left and bottom panels. Scale bar represents 20 μm.
(TIF)

S4 Fig Wg in satellite bouton formation and induction by Sima in glia. (A) Images showing NMJ 6/7 immunostained for Syn (green) and HRP (red). (B) Bar graph shows percentages (mean ± SEM) of satellite boutons: *UAS-wg*, 3.1 ± 1.0%, n = 10; *repo-GAL4/UAS-wg*, 12.7 ± 1.9%, n = 10; *elav-GAL4/UAS-wg*, 9.6 ± 2.3%, n = 6, and *MHC-GAL4/UAS-wg*, 4.4 ± 1.4%, n = 10. (C, E) Images showing NMJ 6/7 immunostained for Wg (green) and HRP (red). (D, F) Bar graph shows averages (mean ± SEM) of normalized Wg to HRP intensities. (D) Wg/HRP ratios: *trh*^*1*^*/trh*^*2*^ (A3), 0.35 ± 0.05, n = 7; *trh*^*1*^*/trh*^*2*^ (A6), 0.18 ± 0.04, n = 8; *repo-GAL4/wg-RNAi trh*^*1*^*/trh*^*2*^ (A3), 0.16 ± 0.04, n = 7, *elav-GAL4/wg-RNAi trh*^*1*^*/trh*^*2*^ (A3), 0.25 ± 0.04, n = 6. (F) Wg/HRP ratios: *UAS-sima*, 0.24 ± 0.06, n = 8; *repo-GAL4/UAS-sima*, 0.54 ± 0.12, n = 9; *elav-GAL4/UAS-sima*, 0.20 ± 0.04, n = 7, and *MHC-GAL4/UAS-sima*, 0.17 ± 0.03, n = 7. Statistical significance was assayed by Mann-Whitney test (n.s., no significance; *, p < 0.05, ***, p < 0.001).
(TIF)

S5 Fig Wg and Sima have minor effects on satellite bouton formation in A6 segment of the *trh* mutant. (A, C) Images showing NMJs of muscle 6/7 immunostained for Syn (green) and HRP (red) in the A6 segment. (B, D) Bar graphs show percentages (mean ± SEM) of satellite boutons in the A6 segment of (B) *repo-GAL4 trh*^*1*^*/trh*^*2*^, *repo-GAL4/UAS-sima trh*^*1*^*/trh*^*2*^, and *repo-GAL4/UAS-wg trh*^*1*^*/trh*^*2*^; and (D) *wg-RNAi trh*^*1*^*/trh*^*2*^, 2.9 ± 1.7%, n = 10 and *repo-GAL4/wg-RNAi trh*^*1*^*/trh*^*2*^, 1.5 ± 1.0%, n = 10. Statistical significance by Mann-Whitney test is shown (n.s., no significance).
(TIF)

S6 Fig Schematic model for satellite bouton induction and crawling defects in the *trh* mutant. (A) In wild-type larvae with normal oxygen supply, the low basal Sima level in glia induces basal Wg expression and secretion to regulate normal synaptic bouton formation. In the *trh* mutant with a defect tracheal system and limited oxygen supply, Sima is upregulated to induce Wg expression and secretion, thus induce satellite bouton formation in the anterior segments of larvae. (B) Wild-type larvae crawl normally with normal sets of synaptic boutons at NMJs of A3 and A6 segments. In the *trh* mutant, the appearance of satellite boutons in the anterior segments (e.g. A3) may be a part of homeostasis for restoring normal synaptic activity while the posterior segments (e.g. A6) were unable to compensate for the reduction of synaptic activity, leading to uncoordinated peristaltic movement.
(TIF)

S1 Movie Forward crawling of *w*^*1118*^. The movie shows freely crawling of *w*^*1118*^ larva in mid-third instar stage for 8.6 seconds with 1x time speed.
(AVI)

S2 Movie Forward crawling of *trh*^*1*^*/trh*^*2*^. The movie shows freely crawling of *trh*^*1*^*/trh*^*2*^ larva in mid-third instar stage for 7.3 seconds with 1x time speed.
(AVI)

S1 Dataset Raw data for Figs 1–8 and S1–S5 Figs. (XLSX)
