# Supplementary figures and images for: Glial response to hypoxia in mutants of NPAS1/3 homolog Trachealess through Wg signaling to modulate synaptic bouton organization
Source: PLoS Genet. 2019 Aug 5;15(8):e1007980. doi: 10.1371/journal.pgen.1007980 (PMC6695205; doi:10.1371/journal.pgen.1007980)

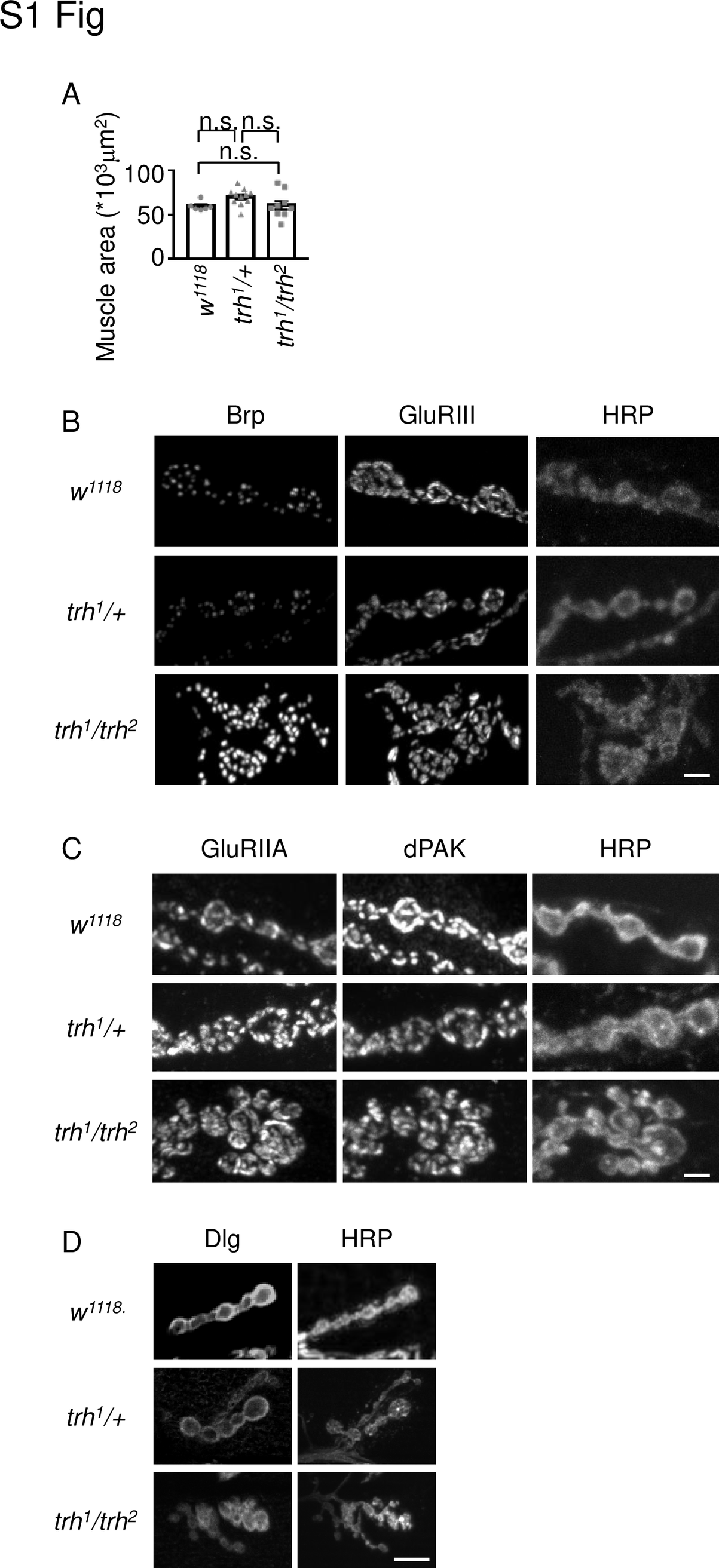

Supplement: S1 Fig — (A) Bar graphs show averages of muscle areas (w1118, 61.2 ± 1.6 x10^3^ μm2, n = 12; trh1/+, 70.0 ± 2.8 x10^3^ μm2, n = 11; and trh1/trh2, 60.5 ± 4.9 x10^3^ μm2, n = 9). (B, C, D) Images showing NMJs of muscle 6/7 immunostained for Brp, GluRIII, and HRP (B), GluRIIA, dPAK, and HRP (C), and Dlg and HRP (D) in w1118, trh1/+, and trh1/trh2. Scale bars are 10 μm. Statistical significance in (A) was assayed by Mann-Whitney test is shown (n.s., no significance). (TIF) [file pgen.1007980.s001.tif]

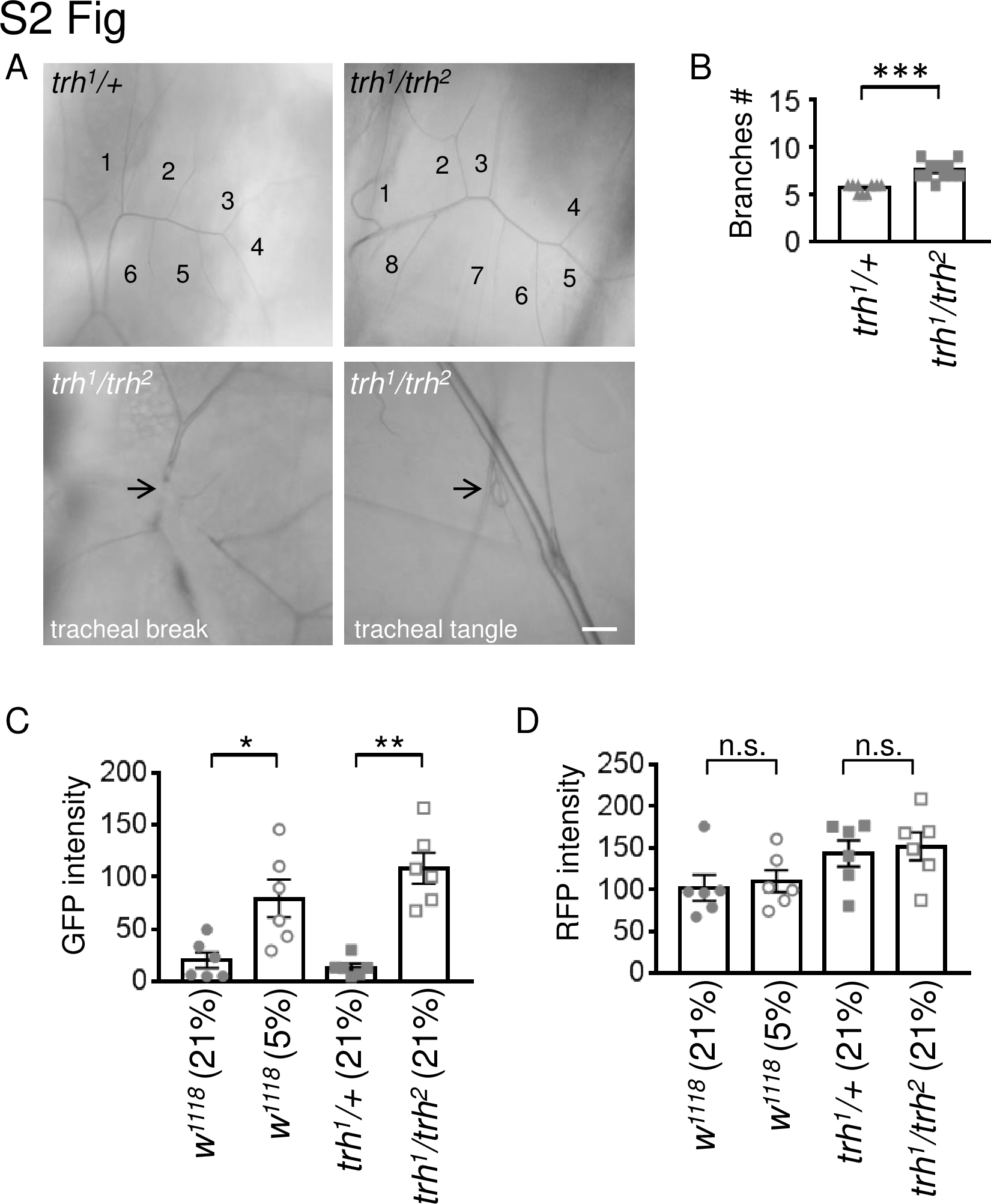

Supplement: S2 Fig — (A) A bright-field view of tracheal dorsal branches of trh1/+ (left top panel) and trh1/trh2 (right top panel), and tracheal dorsal trunks of trh1/trh2 (bottom panels). Numbers denote terminal branches, and arrows indicate a tracheal break (bottom left) and a tracheal tangle (bottom right). Scale bar represents 50 μm. (B) Bar graph shows averages of dorsal terminal branches. (C) Bar graph shows averages of the GFP immunofluorescence intensity (w1118 in 21% O2, 20.3 ± 7.5, n = 6; w1118 in 5% O2, 79.3 ± 18.0, n = 6; trh1/+ in 21% O2, 13.3 ± 3.6, n = 6; trh1/trh2 at 21% O2, 108.2 ± 14.7, n = 6). (D) Bar graph shows averages of RFP immunofluorescence intensity (w1118 in 21% O2, 102.0 ± 15.5, n = 6; w1118 at 5% O2, 109.6 ± 13.1, n = 6; trh1/+ at 21% O2, 143.0 ± 15.7, n = 6; trh1/trh2 at 21% O2, 151.5 ± 16.8, n = 6). Statistical significance by Mann-Whitney test is shown (n.s., no significance; *, p < 0.05; **, p < 0.01; ***, p < 0.001). (TIF) [file pgen.1007980.s002.tif]

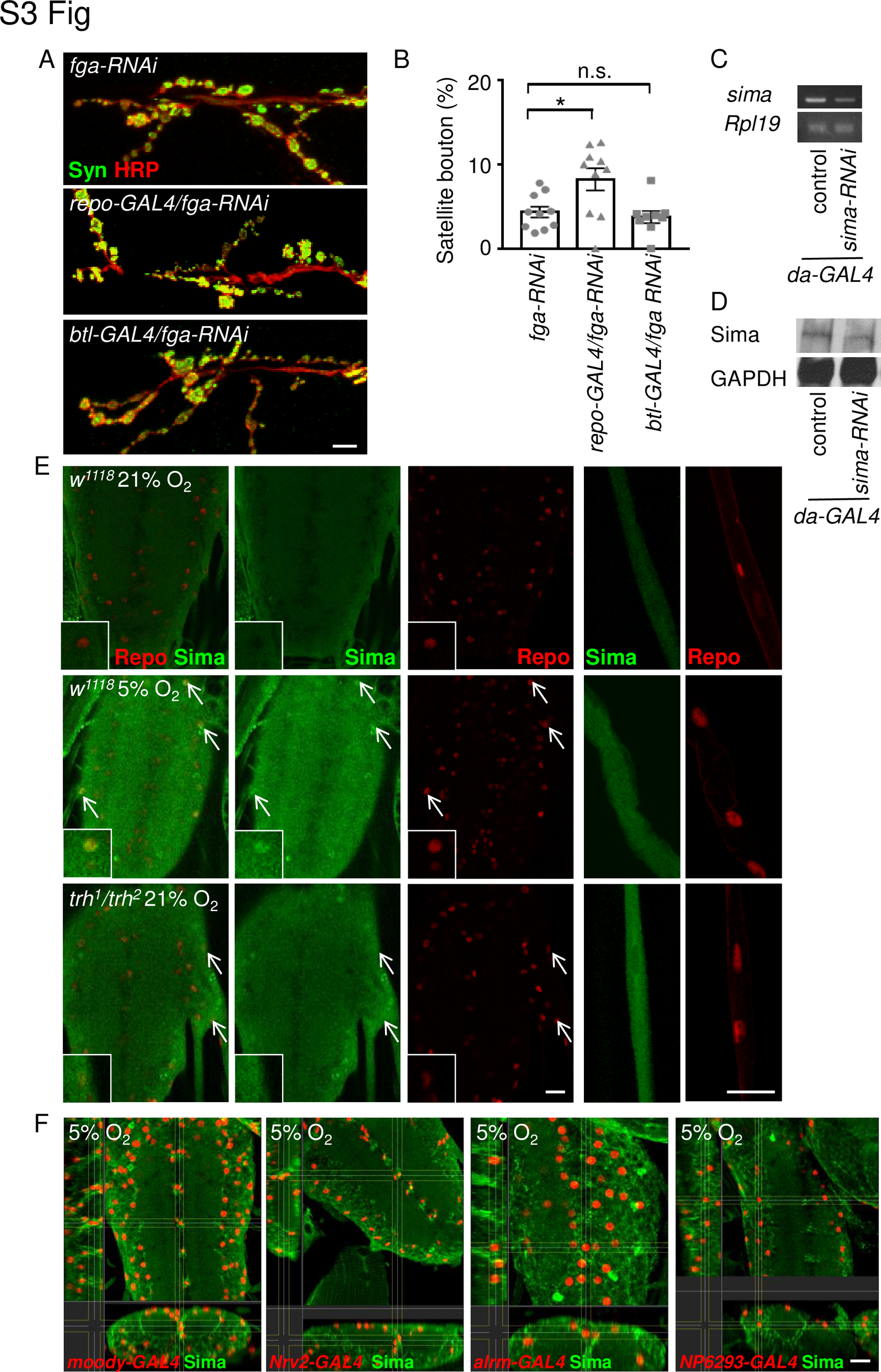

Supplement: S3 Fig — (A) Images showing NMJs from muscle 6/7 immunostained for Syn (green) and HRP (red) in fga-RNAi, and repo-GAL4/fga-RNAi and btl-GAL4/fga-RNAi. (B) Bar graph shows percentages (mean ± SEM) of satellite boutons. Statistical significance was assayed by Mann-Whitney test (n.s., no significance; *, p <0.05). (C, D) RT-PCR for sima mRNA (C) or Western blot for Sima protein (D) expressions in da-GAL4 (control) or da-GAL4/sima-RNAi. The controls Rpl19 mRNA (C) and GAPDH protein (D) are comparable in both genotypes. (E) Images showing central nerve cord (left 3 panels) and peripheral nerve (right 2 panels) immunostained for Sima (green) and Repo (red) for w1118 in 21% O2, w1118 in 5% O2 for 4hrs, and trh1/trh2 in 21% O2. Arrows indicate nuclei of glia with high Sima protein levels. Enlarged images of a nucleus in each condition are shown in left bottom site. (F) Images showing Sima (green) localization in cells of His2Av-mRFP-expressing subperineurial (moody-GAL4), wrapping (Nrv2-GAL4), astrocyte-like (alrm-GAL4) and perineurial (NP6293-GAL4) glia in 5% O2 for 4hrs. Lateral views of the Sima-enriched subtype glia are shown in the left and bottom panels. Scale bar represents 20 μm. (TIF) [file pgen.1007980.s003.tif]

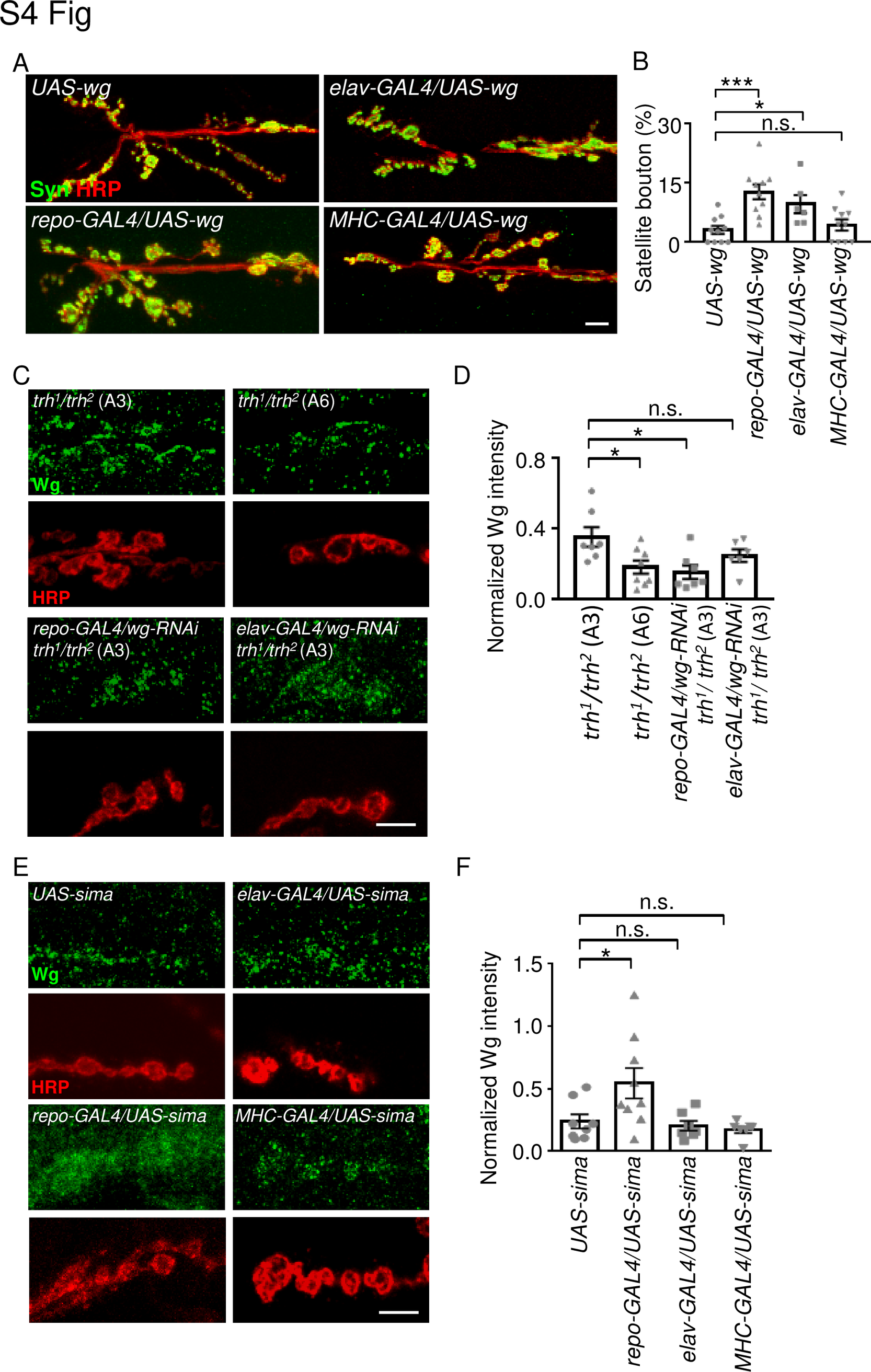

Supplement: S4 Fig — (A) Images showing NMJ 6/7 immunostained for Syn (green) and HRP (red). (B) Bar graph shows percentages (mean ± SEM) of satellite boutons: UAS-wg, 3.1 ± 1.0%, n = 10; repo-GAL4/UAS-wg, 12.7 ± 1.9%, n = 10; elav-GAL4/UAS-wg, 9.6 ± 2.3%, n = 6, and MHC-GAL4/UAS-wg, 4.4 ± 1.4%, n = 10. (C, E) Images showing NMJ 6/7 immunostained for Wg (green) and HRP (red). (D, F) Bar graph shows averages (mean ± SEM) of normalized Wg to HRP intensities. (D) Wg/HRP ratios: trh1/trh2 (A3), 0.35 ± 0.05, n = 7; trh1/trh2 (A6), 0.18 ± 0.04, n = 8; repo-GAL4/wg-RNAi trh1/trh2 (A3), 0.16 ± 0.04, n = 7, elav-GAL4/wg-RNAi trh1/trh2 (A3), 0.25 ± 0.04, n = 6. (F) Wg/HRP ratios: UAS-sima, 0.24 ± 0.06, n = 8; repo-GAL4/UAS-sima, 0.54 ± 0.12, n = 9; elav-GAL4/UAS-sima, 0.20 ± 0.04, n = 7, and MHC-GAL4/UAS-sima, 0.17 ± 0.03, n = 7. Statistical significance was assayed by Mann-Whitney test (n.s., no significance; *, p < 0.05, ***, p < 0.001). (TIF) [file pgen.1007980.s004.tif]

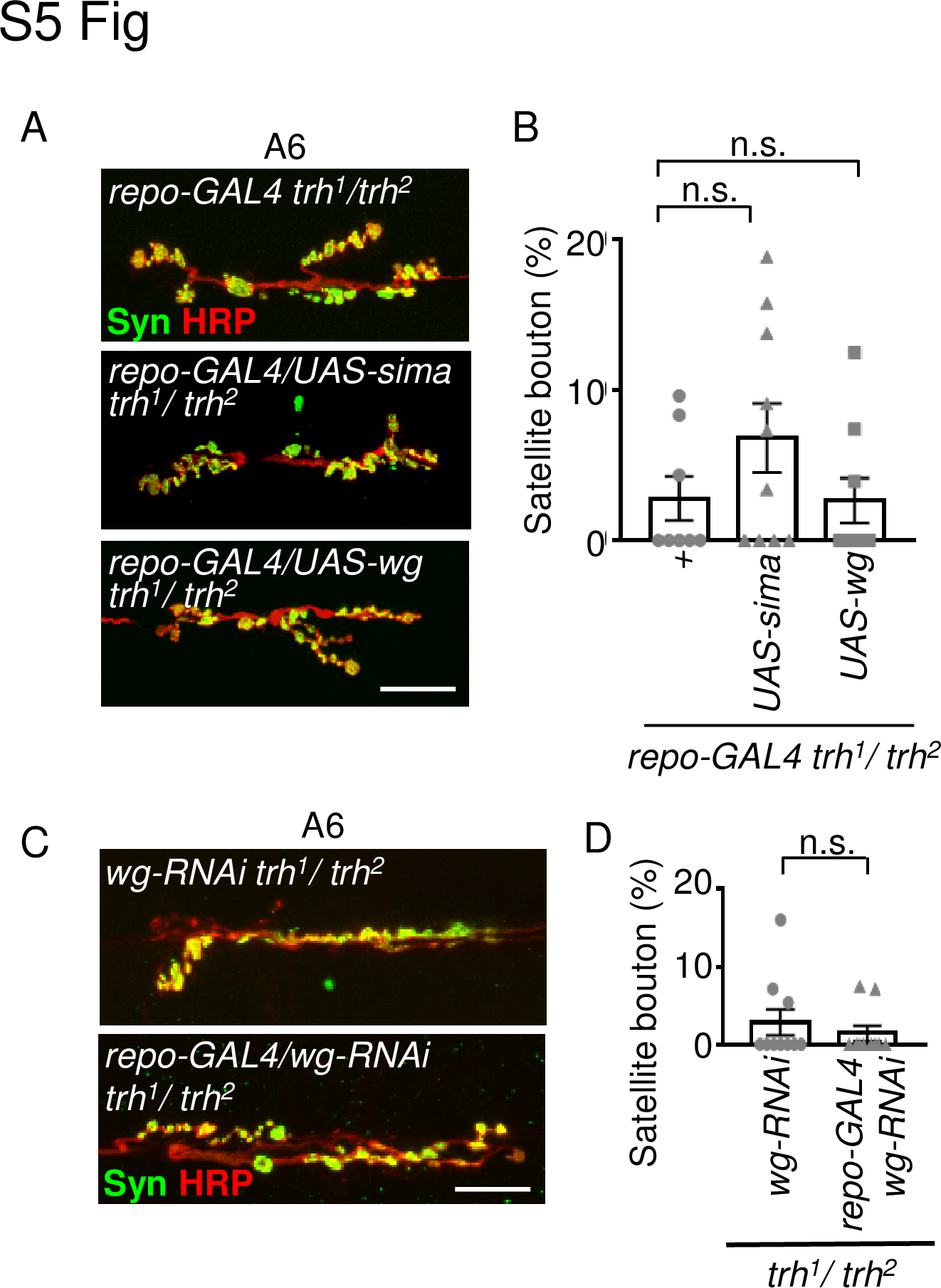

Supplement: S5 Fig — (A, C) Images showing NMJs of muscle 6/7 immunostained for Syn (green) and HRP (red) in the A6 segment. (B, D) Bar graphs show percentages (mean ± SEM) of satellite boutons in the A6 segment of (B) repo-GAL4 trh1/trh2, repo-GAL4/UAS-sima trh1/trh2, and repo-GAL4/UAS-wg trh1/trh2; and (D) wg-RNAi trh1/trh2, 2.9 ± 1.7%, n = 10 and repo-GAL4/wg-RNAi trh1/trh2, 1.5 ± 1.0%, n = 10. Statistical significance by Mann-Whitney test is shown (n.s., no significance). (TIF) [file pgen.1007980.s005.tif]

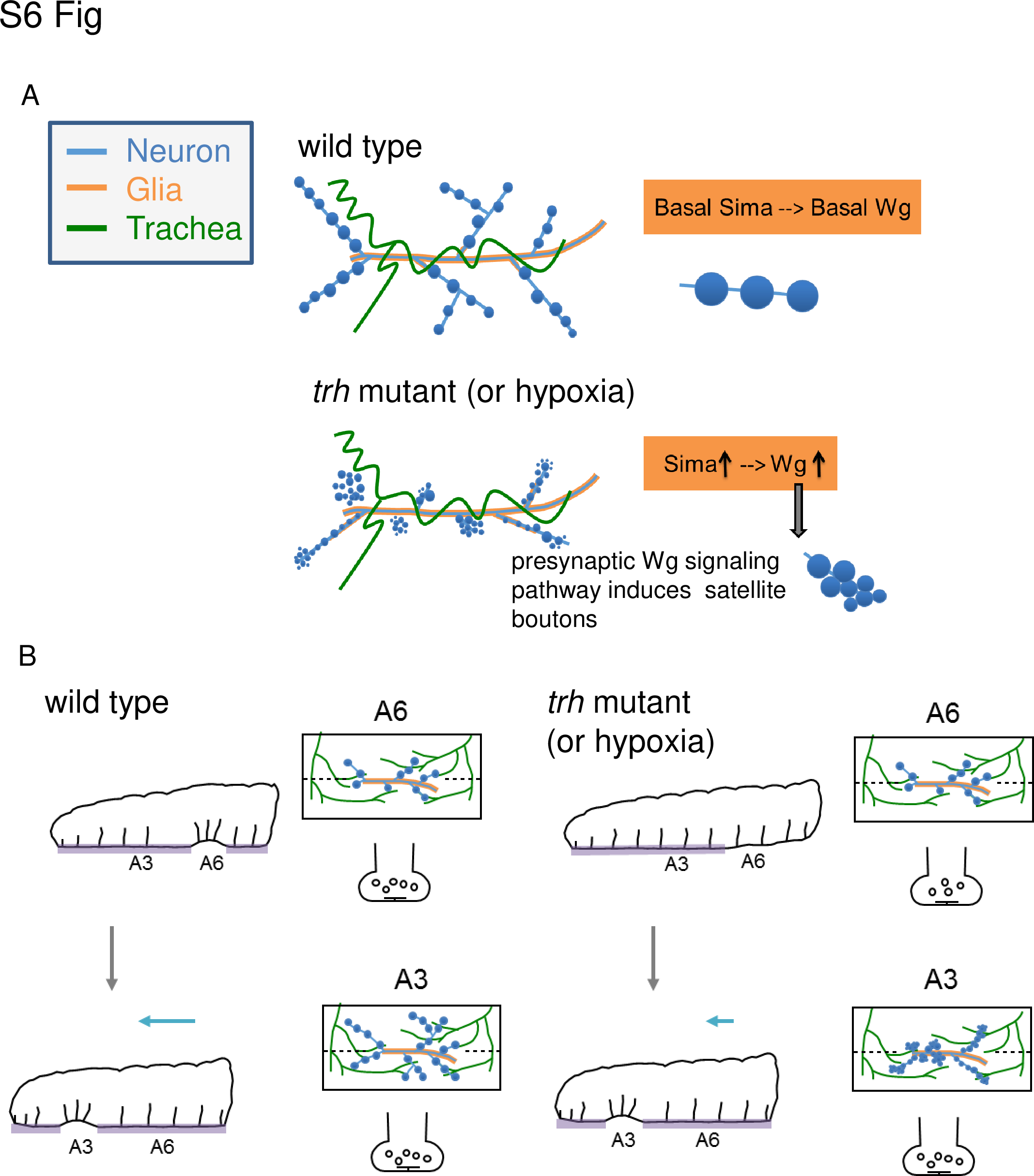

Supplement: S6 Fig — (A) In wild-type larvae with normal oxygen supply, the low basal Sima level in glia induces basal Wg expression and secretion to regulate normal synaptic bouton formation. In the trh mutant with a defect tracheal system and limited oxygen supply, Sima is upregulated to induce Wg expression and secretion, thus induce satellite bouton formation in the anterior segments of larvae. (B) Wild-type larvae crawl normally with normal sets of synaptic boutons at NMJs of A3 and A6 segments. In the trh mutant, the appearance of satellite boutons in the anterior segments (e.g. A3) may be a part of homeostasis for restoring normal synaptic activity while the posterior segments (e.g. A6) were unable to compensate for the reduction of synaptic activity, leading to uncoordinated peristaltic movement. (TIF) [file pgen.1007980.s006.tif]
